# Lipid metabolism as a marker for glioma aggressiveness

**DOI:** 10.1042/BSR20253270

**Published:** 2026-06-03

**Authors:** Hennrique Taborda Ribas, Guilherme Taborda Ribas, Arquimedes Paixão de Santana-Filho, Ana Gabriela Jungles, Clementina Mesaros, Dieval Guizelini, Glaucia R. Martinez, Marina Trombetta-Lima, Guilherme Lanzi Sassaki, Sheila M.B. Winnischofer

**Affiliations:** 1 Postgraduate Program in Biochemistry Sciences, Sector of Biological Sciences, Federal University of Paraná, Curitiba, Brazil; 2 Department of Systems Pharmacology and Translational Therapeutics (SPATT), Perelman School of Medicine, University of Pennsylvania, Philadelphia, PA, U.S.A; 3 Professional and Technological Education Sector, Federal University of Paraná, Curitiba, Paraná, Brazil; 4 Center for Transplantation Sciences, Department of Surgery, Massachusetts General Hospital, Harvard Medical School, Boston, MA, U.S.A.; 5 Biochemistry and Molecular Biology Department, Sector of Biological Sciences, Federal University of Paraná, Curitiba, Brazil; 6 Faculty of Science and Engineering, Department of Pharmaceutical Technology and Biopharmacy, Research Institute of Pharmacy (GRIP), University of Groningen, Groningen, Netherlands; 7 Postgraduate Program in Cellular and Molecular Biology, Sector of Biological Science, UFPR, Curitiba, Brazil

**Keywords:** glioma, Lipid metabolism, lipidomic, plasma membrane

## Abstract

Glioblastoma (GBM) is the most aggressive type of central nervous system tumor. There have been advances in glioma biology understanding; however, the current therapies are still inefficient. Additionally, new molecular markers are being explored for glioma grading, offering potential for novel diagnostic and drug targets. Considering the important role of lipid metabolism in tumorigenesis, understanding the lipid-related pathways in glioma could lead to new important markers. Here, lipid metabolism was analyzed by integrating two different data sources, the transcriptome data from The Cancer Genome Atlas and experimental data from tumor cells, to investigate how lipid metabolism is regulated in glioma. We compared the expression of 743 lipid-related genes in public RNAseq data of glioma patients (*n* = 681) to lipidomic analyses of glioblastoma cell lines (A172, U87MG, and T98G). We identified 29 lipid-related genes correlated to prognosis and constructed a risk signature based on these genes. Extracellular matrix-related genes were positively correlated to the risk score. Our findings revealed that aggressiveness is linked to alterations in membrane composition, characterized by an increase in phospholipids, coupled with a decrease in cholesterol and fatty acid unsaturation levels, which impact membrane physical properties. Modulation of critical signaling lipids, specifically sphingolipids, was also observed. Here, we identify lipid markers that may play a significant role in glioma. The converging axis involving sphingomyelinase and sphingosine-1-phosphate, mediated by the actions of sphingomyelinase SMPD1 and sphingosine-kinase SPHK1, emerged as a pivotal factor in glioma. The present study suggests that lipid membrane composition is pivotal for the GBM aggressiveness.

## Introduction

Gliomas are classified according to their malignancy, with low-grade glioma (LGG) being the least aggressive type [1–3]. Glioblastoma (GBM) is the most aggressive type showing a high lethality. This aggressiveness is explained by its high cellular density, necrosis, and endothelial proliferation, leading to a poor prognosis with a median survival rate of 14 months [4]. Studying GBM could lead to a better biochemical understanding of how lipid mechanisms could be involved in aggressiveness.

Biochemical markers such as choline, an indicator of cellular membrane, could present significant clinical value for glioma diagnosis based on imaging [5–7]. Choline/creatine measurements by magnetic resonance spectroscopy have shown that this ratio rises as the tumoral grade and proliferation index increase [5,8]. The accumulation of choline-containing phospholipids has already been described in several cancers, and its metabolism plays an important pro-tumoral role, supporting a proliferative phenotype [9].

The modulation of cholesterol levels has been described in several studies; however, it is unclear whether a decrease or increase is related to malignancy [10–12]. Cholesterol level modulates biophysical properties of the membrane, with low levels decreasing membrane fluidity and favoring invasion and metastasis of tumor cells [13]. Additionally, squalene epoxidase (SQLE), an important enzyme for cholesterol biosynthesis, is negatively correlated with aggressiveness. Its overexpression leads to the inhibition of migration and invasion in GBM cells [14].

Sphingolipids have been extensively described to modulate tumoral behavior and play an important role in glioma malignancy [15]. Higher levels of ceramide are related to anti-tumoral effects, and several studies have demonstrated that ceramide treatment promotes glioma cell death [16–20]. Ceramide can be generated by either *de novo* synthesis or by degradation of other sphingolipids, such as sphingomyelin, which is converted to ceramide by sphingomyelinase [21]. On the other hand, sphingosine-1-phosphate (S1P) has been shown to promote pro-tumoral effects and is correlated to glioma malignancy [15]. The present study explores the entire lipid metabolism in glioma to investigate which lipids are related to aggressiveness.

## Clinical significance

The present study holds significant importance in unraveling the lipid metabolism linked to glioma by integrating clinical and experimental data. Exploring the impact of lipid profiles on glioma aggressiveness unveils potential biomarkers, diagnostic tools, and therapeutic targets. The presented NMR-derived lipidomic data identifies potential markers of aggressiveness that could serve as a tool for non-invasive magnetic resonance spectroscopy in patients. These metabolic signatures can be non-invasively monitored over time, aiding in treatment monitoring and prognosis assessment. Furthermore, the exploration of sphingolipid signaling as a key player in glioma progression provides valuable insights into the underlying mechanisms of this highly aggressive tumor. Understanding the lipid-related pathways and identifying relevant markers for glioma proliferation can ultimately contribute to more effective treatments and improved patient outcomes.

## Materials and methods

### Bioinformatic analysis

The gene set ‘REACTOME_METABOLISM_OF_LIPIDS’ was obtained from the Human Molecular Signatures Database (MSigDB), and 743 lipid metabolism-related genes were extracted. The TCGA RNAseq normalized gene expression data of 743 lipid-related genes from 681 glioma (LGG and GBM) patients were collected from FireBrowse (http://firebrowse.org/). The RNAseq normalized gene expression data from CGGA (*n* = 984) were collected from the GlioVis data portal for visualization and analysis of brain tumor expression datasets (http://gliovis.bioinfo.cnio.es/) [22]. The expression was compared between groups by calculating *t*-test using the statistical function of the scientific computation library SciPy in Python. The data regarding survival and PTEN status were collected from the Gliovis website (http://gliovis.bioinfo.cnio.es/), and the survival analysis was calculated by using the KaplanMeierFitter function of the Lifelines library in Python. Further, the up- and down-regulated genes were separated according to their mean values. The modulated genes were subjected to Gene Ontology (GO) and function cluster annotation analysis using DAVID Bioinformatics Resources (http://david.abcc.ncifcrf.gov/home.jsp) to determine significantly enriched genes. All the graphs were generated in Python by using the data visualization library Seaborn.

To identify genes with significant prognostic value, gene expressions that showed statistical differences between LGG and GBM underwent a univariate Cox analysis, where those with a *P*-value <0.001 were considered prognostically relevant. Subsequently, these chosen genes were integrated into a Cox regression model utilizing the least absolute shrinkage and selection operator technique. The resulting coefficients for each gene were employed to compute the risk score: risk score = ∑(Coefi × Xi). The threshold for categorizing individuals into the high-risk and low-risk groups was set at the median risk score. The difference in survival outcomes between the high-risk and low-risk groups was evaluated through the creation of survival curves, followed by the application of the log-rank test. Different false discovery rate (FDR) cutoffs were applied in independent analyses to separate up- and down-regulated genes, allowing for a more comprehensive identification of deregulated genes.

### Cell culture

The A172, U87MG, and T98G human GBM cell lines were kindly provided by Professor Mari C. Sogayar, University of São Paulo. Cells were cultured in DMEM culture medium supplemented with 10% fetal bovine serum and 25 μg/ml gentamicin (Gibco) at 37°C in a humid atmosphere containing 5% CO_2_ and maintained at approximately 80% confluency. For growth curves, 1 × 10^4^ cells were seeded in 48-well plates. Triplicates were collected on days 1, 3, 5, and 7, using trypsin, fixed in 3.7% formaldehyde, and counted using the Neubauer chamber. The graph was generated in Python by using the data visualization library Seaborn.

### Lipid extraction

Lipid extraction for NMR analysis was performed as previously described [23]. A total of 2.3 × 10^6^ cells were seeded in a 150 mm plate (eight plates) for 24 h, then collected using 0.1% trypsin solution containing 1 mM EDTA and centrifuged at 2000×***g*** for 5 min (4°C). The cell pellet was washed thrice with phosphate buffer, and the supernatant was discarded. The cell pellets were subjected to lysis by freezing into liquid nitrogen and returned to a 37°C water bath several times. After being freeze-dried overnight, 10 mg of each cell line was weighed and transferred to a glass tube with a screw Teflon cap (volume of 4 ml). The pellet was extracted using CHCl_3_:MeOH 1:1 (v/v) (1 ml), vortexed for 1 min, and then kept for 10 min at room temperature in an ultrasonic bath. The supernatant was collected, and the solvent dried under a stream of nitrogen.

For LC-MS analysis, we applied a butanolic extraction procedure described by Baker et al. [24]. In brief, 1.5 × 10^5^ cells seeded in six wells were scraped directly into 1 ml of 80% methanol containing internal standards, 20 μl of SPLASH^®^ LIPIDOMIX^®^ (Avanti Polar Lipids #330707), and 20 μl of Cer/Sph Mixture I (Avanti Polar Lipids #LM6002) diluted 1:1 (v:v) with 20 ng of each Ceramide-D7 C16 Ceramide-d7 (d18:1-d7/16:0) (#860676); C18 Ceramide-d7 (d18:1-d7/18:0) (#860677); C24:1 Ceramide-d7 (d18:1-d7/24:1) (#860679); and C24 Ceramide-d7 (d18:1-d7/24:0) (#860678).

Extractions were mixed with 120 μl of a buffer containing 200 mM citric acid and 270 mM disodium hydrogenphosphate (pH 4). Extraction was performed with 2 ml of 1-butanol and 1 ml of water-saturated 1-butanol. The recovered butanol phase was evaporated to dryness under reduced pressure. The residue was redissolved in 100 μl MTBE:methanol (1:3, v/v). From each sample, 40 μl were pooled to generate a quality control (QC) sample, which was injected after every 10 analytical runs. The remaining volume (∼60 μl) was transferred to amber glass HPLC vials for LC-MS analysis. Extraction blanks and internal standard-only controls were processed in parallel to monitor background and recovery efficiency.

### NMR spectroscopy

CDCl_3_ was purchased from Cambridge Isotope Laboratories, Inc. (Miami, U.S.A.) and from Sigma-Aldrich (St. Louis, MO). The samples were deuterium exchanged by repeated dissolution in MeOD-D_2_O (2:1). Both were freeze-dried overnight. Their spectra were obtained in CDCl_3_-MeOD (3:1, 600 μl) at 30°C, using methylsulfonylmethane (MSM) as an internal standard (δ = 3.03 ppm). Spectra were obtained on a Bruker 600 MHz ASCEND equipped with a QXI probe (Bruker Biospin, Germany). 1D ^1^H-NMR was carried out using enough scans to give a signal/noise (S/N) ratio of at least 2000/1 (90° pulse, relaxation delay = 4.0 s, number of time domain points = 65,536, spectral width = 10.6541 ppm, and acquisition time = 7.7 s). Experiments were performed without tube rotation and with the MSM or TSP signal at a medium half-line width ranging from 0.6 to 1.0 Hz. For multivariate analysis and estimation of metabolite concentrations, the spectra were acquired using the noesygppr1d.2 pulse sequence, with 128 transients and a delay of 10.0 s (2.0 s in the analysis of lipid samples). 2D NMR experiments were carried out using multiplicity-edited ^1^H-^13^C HSQC, heteronuclear correlation via double INEPT transfer with decoupling during acquisition, and using trim pulses in INEPT transfer with multiplicity editing during the selection step (hsqcedetgpsp.3).

### Lipid analysis by LC-MS

Lipids were separated using an Accucore C18 HPLC column (2.1 × 100 mm, 2.6 μm, #17126-102130) (Thermo Scientific, Waltham, MA) at 35°C on an UltiMate 3000 quaternary UHPLC equipped with a refrigerated autosampler (10°C). Solvent A was 1/1 (v/v) acetonitrile/water with 10 mM ammonium formate and 0.1% formic acid. Solvent B was 10/88/2 acetonitrile/isopropanol/water with 2 mM ammonium formate and 0.02% formic acid. Flow rate was 0.4 ml min^−1^. Flow gradient conditions were as follows: 0 min, 90% A; 1 min, 90% A; 4 min, 60% A; 12 min, 25% A; 21 min, 1% A; 24 min, 1% A; 24.1 min, 90% A; 28 min, 90% A. Samples were analyzed using the Thermo Scientific™ Orbitrap™ IQ-X™ Tribrid™ Mass Spectrometer operated in positive ion mode, then in negative ion mode. Two-microliter injections were made. Column effluent was diverted to the IQX from 2 to 23 min and to waste for the remaining time of the run. ESI operated in both positive and negative ionization modes. In positive mode, the spray voltage was 4000 V, capillary temperature 285°C, sheath gas flow 50 (arbitrary units), auxiliary gas flow 15, and spray current 100 μA. In negative mode, the spray voltage was 3500 V, with sheath and auxiliary gas flow rates of 55 and 20, respectively. All other source parameters were optimized to ensure stable spray performance and consistent ion transmission throughout the analytical run.

Lipid species were identified using LipidSearch (Thermo Fisher Scientific). Features were retained if they showed a coefficient of variation (CV) <20% in pooled quality control (QC) samples and a QC-to-blank intensity ratio >5. Data were log_2_-transformed prior to univariate analysis and log_2_-transformed with Pareto scaling for multivariate analyses, performed in MetaboAnalyst 6 (https://www.metaboanalyst.ca/). Univariate comparisons were conducted using two-sided *t*-tests, and *P*-values were adjusted for multiple testing using the Benjamini–Hochberg FDR method. Further statistical analyses and visualizations were performed in Python (v3.12) using the SciPy, statsmodels, scikit-learn, and matplotlib libraries.

### Simulation

The membrane compositions used for molecular dynamics simulations were constructed directly from experimentally determined lipid parameters obtained by NMR analysis. Specifically, the relative phosphatidylcholine (PC) abundance and cholesterol content were incorporated as molar percentages, and the experimentally measured unsaturation ratio was used to subdivide the PC fraction into more unsaturated (modeled as DOPC) and less unsaturated (modeled as DIPC) components. This approach allowed each simulated membrane to quantitatively reflect the lipidomic profile of the corresponding cell line. The GROMACS 2019.1 MD [25,26] engine and MARTINI 2.2 [27] coarse-grain forcefield were used for simulations. Three membranes (15 × 15 × 10 nm) included polarizable MARTINI water and 150 mM NaCl and were constructed using the insane package [28]. Each membrane was energy-minimized with steepest descent, following 50 ns of equilibration at 310 K (with a coupling constant of 1.0 ps) using a 10 fs timestep and constant temperature and pressure. Pressure was maintained at 1 bar with a Berendsen barostat (3 × 10^−4^ bar^−1^, 12 ps). Production simulations were performed for 20 μs in triplicate, with each replicate initiated from different velocities. Temperature was maintained the same as for equilibration, while semi-isotropic Parrinello–Rahman pressure coupling (1 bar, 3 × 10^−4^ bar^−1^, 12 ps) was used to maintain pressure [29]. All simulations were conducted with a timestep of 20 fs, according to standard MARTINI configuration recommendation [30]. The screenshot images were generated using the 3D viewer MOL* [31] from the RCSB PDB (https://www.rcsb.org/) [32,33].

Biophysical properties were analyzed using ROH, GL1, and GL2 beads as references for cholesterol and phospholipids, respectively. Reported values include SEM between replicates. The area per lipid (APL) was calculated according to its definition: the cross-sectional area (AXY) of the whole system along the bilayer surface plane (*XY*-plane), divided by half the total number of lipids (NL) present in the bilayer, i.e., AL = AXY/(NL/2). Membrane thickness, flip-flop rate, and self-diffusivities were calculated using the lipyphilic package [34].

### Quantitative gene expression assays

Reverse transcription was performed with 1 μg total RNA and random primers with ImProm II Reverse Transcriptase Kit (Promega, Madison, USA). qPCR was carried out using the GoTaq® Real-Time PCR Systems (Promega) on a StepOne Plus thermal cycler (Applied Biosystems), according to the recommended protocol. Data were normalized according to Vandesompele et al. [35], using as reference genes GAPDH, HMBS, and HPRT, with the HMBS being selected as the best normalizer.

Primer sequences used were SPHK1 sense: 5′-ATTATGCTGGCTATGAGCAGG-3′; SPHK1 antisense: 5′-TGCAGAGACAGCAGGTTCAT-3′; SPHK2 sense: 5′-CCAGACAGAACGACAGAACCAC-3′; SPHK2 antisense: 5′-CTCCCGAGACCGTGACGATG-3′; SMPD1 sense: 5′-ACCGAATTGTAGCCAGGTATGA-3′; SMPD1 antisense: 5′-AGAAGACCTCAAATTCATCCACA-3′; GAPDH sense: 5′-ACCCACTCCTCCACCTTTGA-3′; GAPDH antisense: 5′-CTGTTGCTGTAGCCAAATTCGT-3′; HPRT sense: 5′-GAACGTCTTGCTCGAGATGTGA-3′; HPRT antisense: 5′-TCCAGCAGGTCAGCAAAGAAT-3′; HMBS sense: 5′-TGGACCTGGTTGTTCACTCCTT-3′; and HMBS antisense: 5′-CAACAGCATCATGAGGGTTTTC-3′.

### Statistical analysis

All experimental data were obtained with three independent experiments. The ANOVA was complemented with Tukey’s test, and the calculations were performed with Python software using stastmodel module. All statistical tests were considered at the predetermined level of significance of 5%. All the graphs were generated in Python by using the data visualization library Seaborn.

## Results

### Identification of lipid metabolism-related genes with prognostic value in glioma

**Figure 1:**
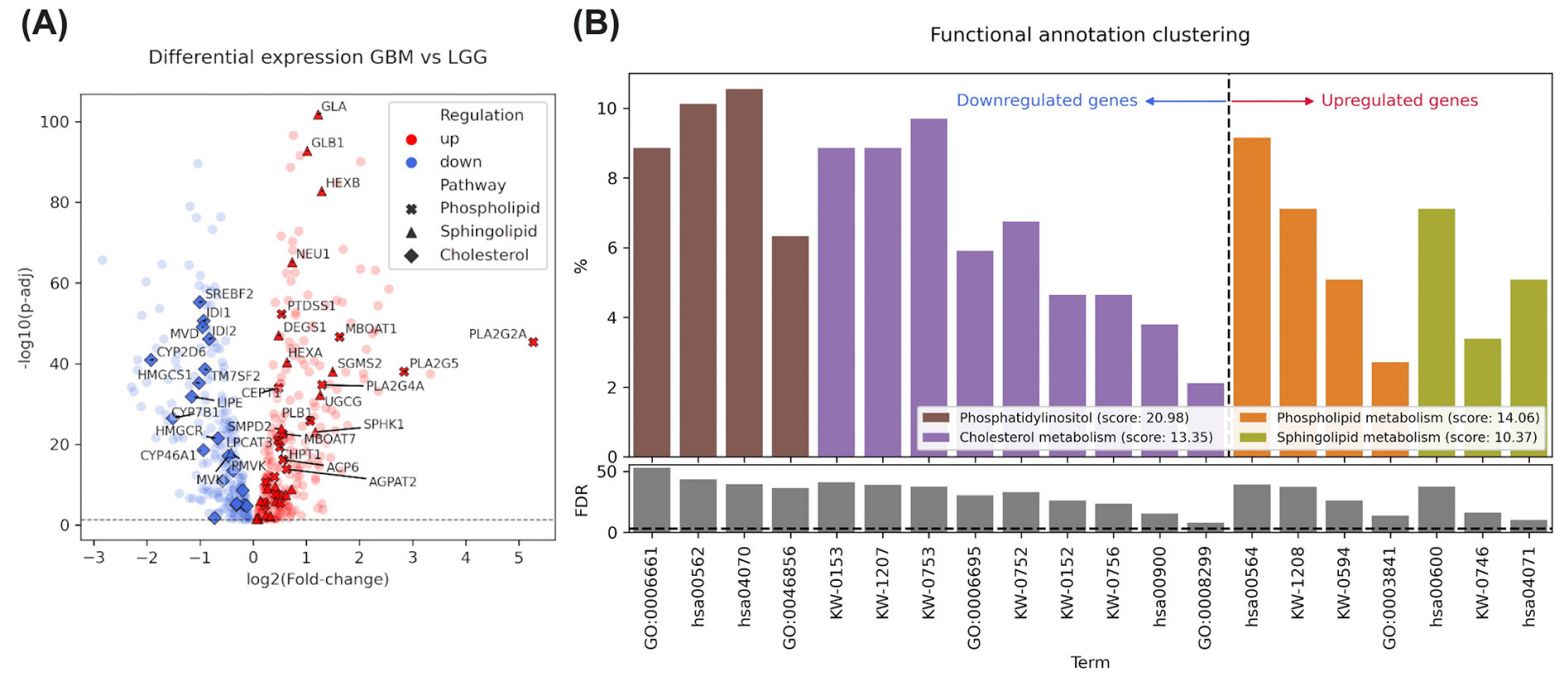
Lipid metabolism pathways related to glioma aggressiveness (**A**) Lipid metabolism-related DE genes (LGG versus GBM) are represented on volcano plot; genes involved in the most deregulated pathways are highlighted. (**B**) Enrichment analysis, identifying related genes by measuring the similarity of their global annotation profiles of down- and up-regulated lipid metabolism-related genes using a *t*-test (*P <*0.05) comparing expression data of GBM to LGG patients (*n* = 681); the genes were classified into three functional gene groups based on the functional similarity scores. Statistical values of −ln(FDR) to rank their biological significance are shown. GO:0006661: Phosphatidylinositol biosynthetic process; hsa00562: Inositol phosphate metabolism; hsa04070: Phosphatidylinositol signaling system; GO:0046856: phosphatidylinositol dephosphorylation; KW-0153: Cholesterol metabolism; KW-1207: Sterol metabolism, KW-0753: Steroid metabolism; GO:0006695: Cholesterol biosynthetic process; KW-0752: Steroid biosynthesis; KW-0152: Cholesterol biosynthesis; KW-0756: Sterol biosynthesis; hsa00900: Terpenoid backbone biosynthesis; GO:0008299: Isoprenoid biosynthetic process; hsa00564: Glycerophospholipid metabolism; KW-1208: Phospholipid metabolism; KW-0594: Phospholipid biosynthesis; GO:0003841: 1-acylglycerol-3-phosphate O-acyltransferase activity; hsa00600: Sphingolipid metabolism; KW-0746: Sphingolipid metabolism; hsa04071: Sphingolipid signaling pathway.

To evaluate how lipids could be related to glioma aggressiveness, we first assessed the expression data of 743 lipid-related genes in public RNAseq data from TCGA of glioma patients (*n* = 681). We compared the expressions between LGG and GBM by *t*-test and select the genes that are up- and down-regulated (*P* <0.05; −log10(*P*-value) < 1.3) in the most aggressive subtype to perform enrichment analysis (Figure 1A,B). High-grade glioma showed higher expression of genes related to the synthesis of membrane lipids such as glycosphingolipids, phosphatidylcholine, and the signaling lipids, sphingolipids. Whereas reduced expression of genes related to phosphatidylinositol and cholesterol synthesis was present in GBM.

**Figure 2:**
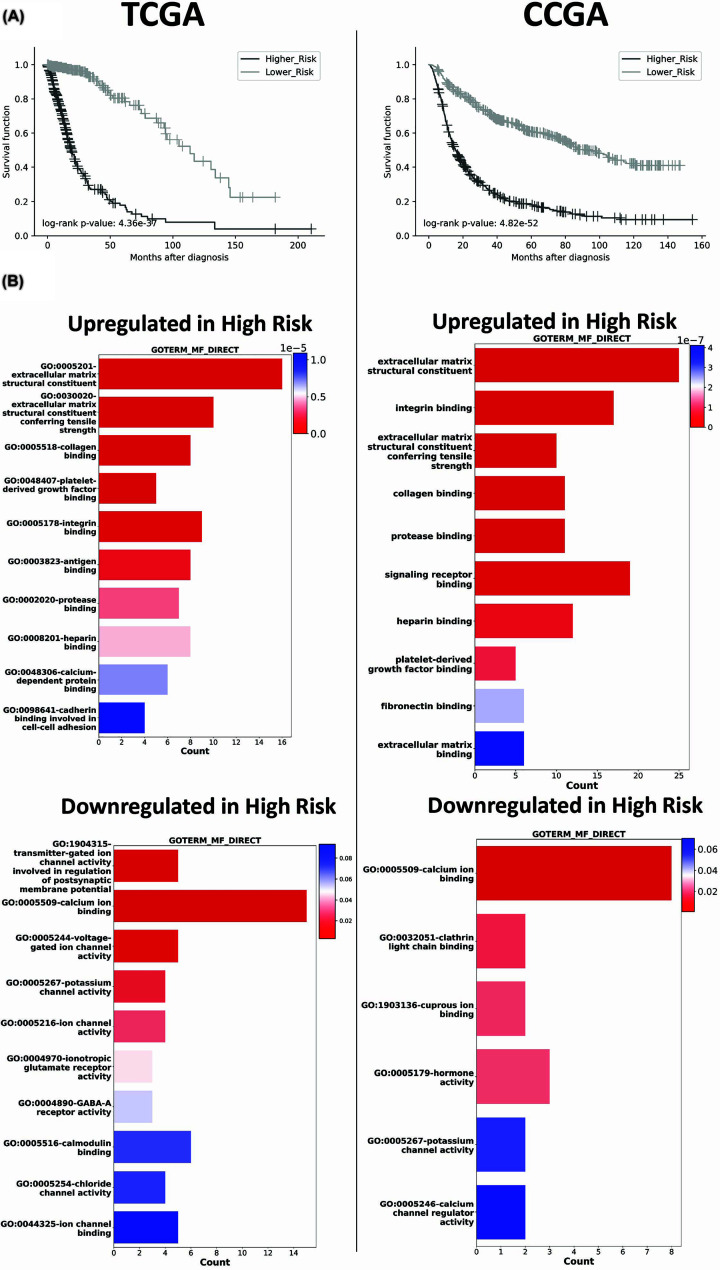
Lipid-associated risk score establishment (**A**) Survival curves of patient according to the lipid-associated risk score using the median risk value as cutoff. (**B**) Enrichment analysis, using molecular functional (MF) annotation from GO, of down- and up-regulated genes using *P <*0.05 and |logFC| > 2.5 comparing expression data of high-risk low-risk patient groups. Statistical values to rank their biological significance are represented by gray scale.

To evaluate the relationship between lipid metabolism-related genes and glioma patients’ survival, we first collected the most differently expressed (DE) genes (*P <*0.05, |logFC| >1) to further perform univariate Cox regression analysis. We identified 109 DE genes between LGG and GBM samples, and after univariate Cox regression analysis, 29 genes were significantly associated with patient prognosis (*P <*0.001). Subsequently, the chosen genes were integrated into a Cox regression model, resulting in coefficients for each gene, which were then employed to calculate the risk score (Supplementary Table S1). The median risk score was used as a cutoff to separate patients into the groups, high-risk group and low-risk group. Consistently, Kaplan–Meier analysis showed a significant difference in overall survival between high-risk and low-risk patients (Figure 2A). We further validated the coefficient of the lipid genes to predicted risk in patients using a different transcriptome database, CGGA. Interestingly, we could also evidence a significant difference in overall survival between high-risk and low-risk patients in CGGA patients (Figure 2A).

Furthermore, we analyzed DE genes related to risks by comparing high- to low-lipid-related risk groups and performed an enrichment analysis of significant gene expressions (*P* <0.05, |logFC| > 2.5). Consistently, as shown in Figure 2B,C, in both databases (TCGA and CGGA), genes related to the extracellular matrix (ECM) and its regulation are up-regulated in high-risk patients, while genes related to ion transportation are down-regulated.

Taken together, the data indicates that there is a cross-talk between membrane composition and ECM and ion transportation, which could play an important role in the tumor aggressiveness phenotype.

### Cholesterol, phospholipid, and sphingolipid metabolisms are modulated in high grades gliomas

**Figure 3:**
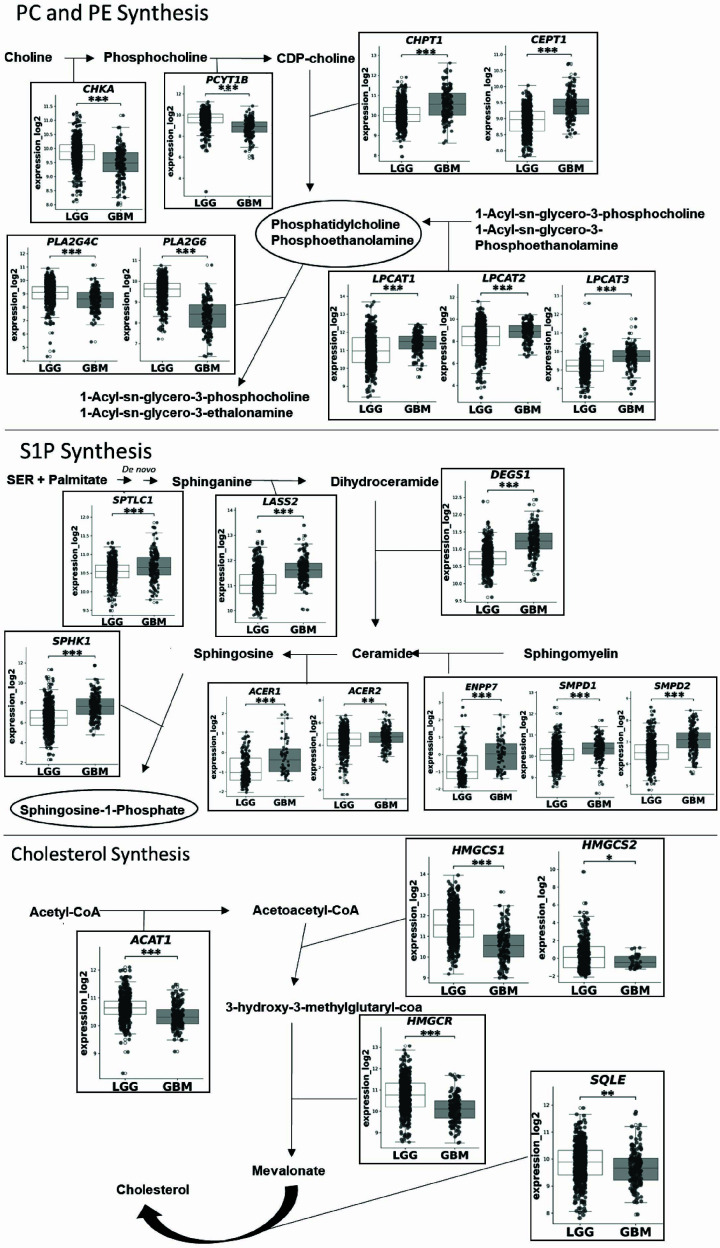
Lipid metabolism pathways related to glioma aggressiveness Representation of the three most important lipid metabolism pathways with the expressions of the genes that are being modulated according to aggressiveness, *t*-test was used to compare between groups *P <0.05; **P <0.01;***P <0.001 (*n* = 681).

To better understand how lipid metabolism pathways changed according to the aggressiveness phenotype, we further analyzed the differentially expressed lipid metabolism-related genes. Important genes for phosphatidylcholine and phosphoethanolamine synthesis are more expressed in GBM and related to lower survival rates, whereas, in LGG, higher expression of genes involved with their degradation is presented (Figure 3 and Supplementary Figure S1). Although it has been shown that the two initial steps of phospholipid biosynthesis responsible for converting choline to CDP-choline are related to a less aggressive type of glioma, as shown by the higher expression of *CHKA* and *PCYT1B* in LGG as well as by the higher survival rate in patients overexpressing these genes (Figure 3 and Supplementary Figure S1). The data also suggested that GBM might have higher levels of the pro-tumoral sphingolipid S1P generated from different sources such as *de novo* synthesis and sphingomyelin degradation (Figure 3). Taken together, these findings indicate that increased expression of genes associated with phosphatidylcholine, phosphatidylethanolamine, and S1P metabolism is associated with glioma aggressiveness.

On the other hand, our analysis indicates that cholesterol metabolism is differentially regulated in LGG. Several genes involved in the mevalonate pathway are differentially expressed in this subtype. Notably, HMGCR and SQLE, two key enzymes regulating cholesterol biosynthesis, are associated with improved survival, suggesting a relationship between cholesterol pathway gene expression and clinical outcome in LGG (Figure 3 and Supplementary Figure S1). The phosphatidylinositol pathway was also found to be more transcriptionally active in LGG (Figure 3).

Since PTEN mutation could be related to GBM, we assessed whether the phosphatidylinositol pathway modulation would be due to a higher PTEN mutation prevalence in this group. Indeed, PTEN mutation is more incident in GBM patients as shown in Supplementary Figure S2; furthermore, comparing patients with PTEN mutant (PTENmut) with PTEN WT shows that mutation in PTEN is correlated to lower expression of genes involved in the phosphatidylinositol (PI) pathway (Supplementary Figure S2). Interestingly, PTENmut patients also showed lower expression of genes related to the cholesterol pathway, suggesting that also in the more aggressive phenotype, by considering PTEN status, the cholesterol pathway is also down-regulated. (PTEN mutation is related to a worse prognosis; therefore, the cholesterol pathway is down-regulated in a more aggressive phenotype not only considered by tumor grade but also by the PTEN status).

Importantly, when restricting the analysis to IDH-wild-type patients, the differential expression of lipid metabolism-related genes between LGG and GBM remained consistent. Survival analyses within this subgroup showed similar trends (Supplementary Figures S3 and S4), supporting the robustness of these associations independent of IDH mutation status.

### Cholesterol and phospholipids metabolisms are modulated in glioblastoma cells with different aggressiveness phenotypes

**Figure 4:**
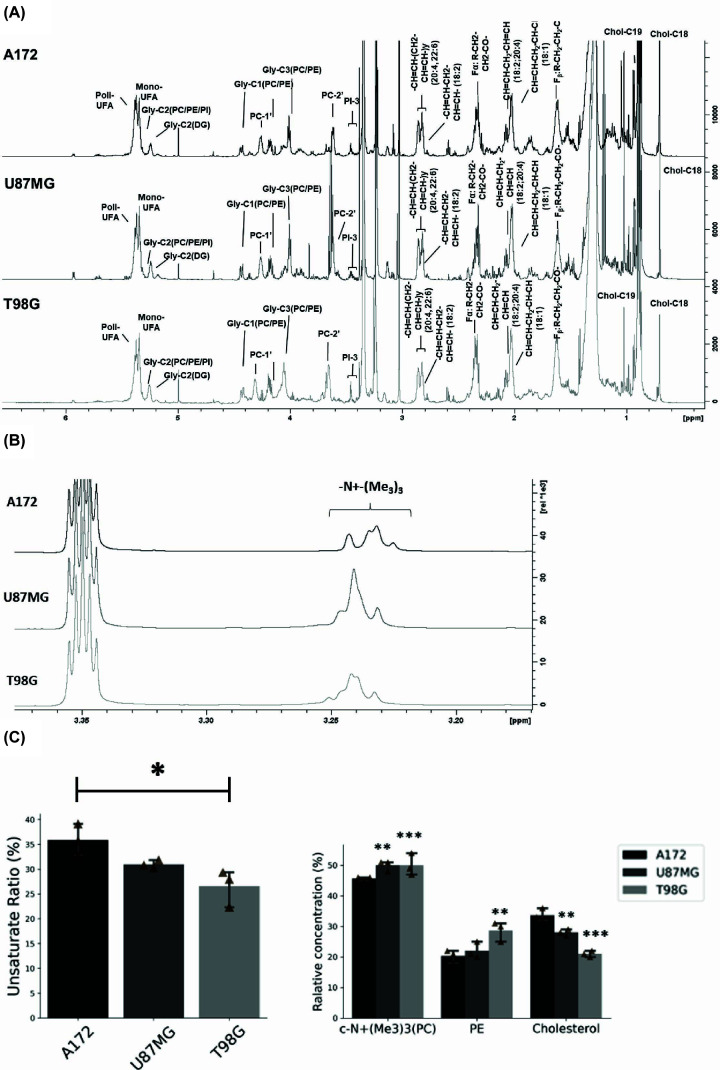
Lipid profile related to aggressiveness *in vitro* (**A**) 1H NMR spectra of the lipid extract of the GBM cells. (**B**) 1H NMR spectra showing the different profiles of lipids containing choline between the cells. (**C**) The relative quantification of the main lipid groups using MSM as an internal standard: data related to three independent experiments. Statistical analysis was performed using one-way ANOVA followed by Tukey's multiple comparisons test to compare A172 with U87MG and T98G. Data are represented as mean ± SEM. ** represents *P <*0.01; *** represents *P <*0.001. For more detailed assignments, see Supplementary Table S2.

To investigate the lipid metabolism in the aggressiveness phenotype and confirm the clinical data, we used three PTEN mut glioblastoma cell lineages with different pre-described tumorigenic phenotypes (A172, U87MG, and T98G) [36,37]. A172 is the least aggressive cell line with the slowest proliferation rate, reaching the saturation density of 10.24 × 10^4^ cells/cm^2^ (SD = 1.22 × 10^4^) at the 9th day, while U87MG presented a saturation rate of 29.7 × 10^4^ cells/cm^2^ (SD = 1.61 × 10^4^) (Supplementary Figure S5). The most proliferative cell line, T98G, reaches the stationary phase in 7 days with a similar saturation density as U87MG, 28.7 × 10^4^ cells/cm^2^ (SD = 1.28 × 10^4^). Then, we performed a lipidomic analysis of the lipid extract of those cells by using standardized ^1^H NMR spectra (Figure 4A). The ^1^H NMR analysis showed that the three glioblastoma linages presented similar lipid profiles, with the exception of the phosphocholine-related lipid profile (Figure 4B). That suggests different levels of choline, phosphocholine, and phosphatidylcholine among the cell lines. Furthermore, quantification of this data showed that A172, which presents the less aggressive phenotype, contains lower levels of total phosphocholine-related lipids, ethanolamine lipids, and higher levels of cholesterol (Figure 4C). These results confirm the bioinformatics data, suggesting that phospholipid and cholesterol play opposite roles in glioblastoma aggressiveness.

### Membrane fluidity could be modulated according to the glioblastoma aggressiveness

We also observed different levels of fatty acid unsaturation rates according to the aggressiveness phenotype in the glioblastoma cells (Figure 4C). The less aggressive cell line, A172, presented higher levels of unsaturated to saturated fatty acids; furthermore, the unsaturated ratio decreases as the aggressiveness increases. To further assess the relation between malignancy and fatty acid unsaturation, we also assessed the expression of genes related to fatty acid biosynthesis in patients with different malignant grades (Supplementary Figure S6A). Interestingly, high-grade patients showed lower expression of desaturase genes, also suggesting that decreased levels of unsaturated fatty acids are related to malignancy. Since fatty acid acyl chain length also impacts the membrane fluidity, we evaluated the expression of genes related to fatty acid elongation. Higher expression of these genes associated with lower expression of desaturase genes suggests that biophysical properties of membranes change according to aggressiveness in gliomas (Supplementary Figure S6B).

**Figure 5:**
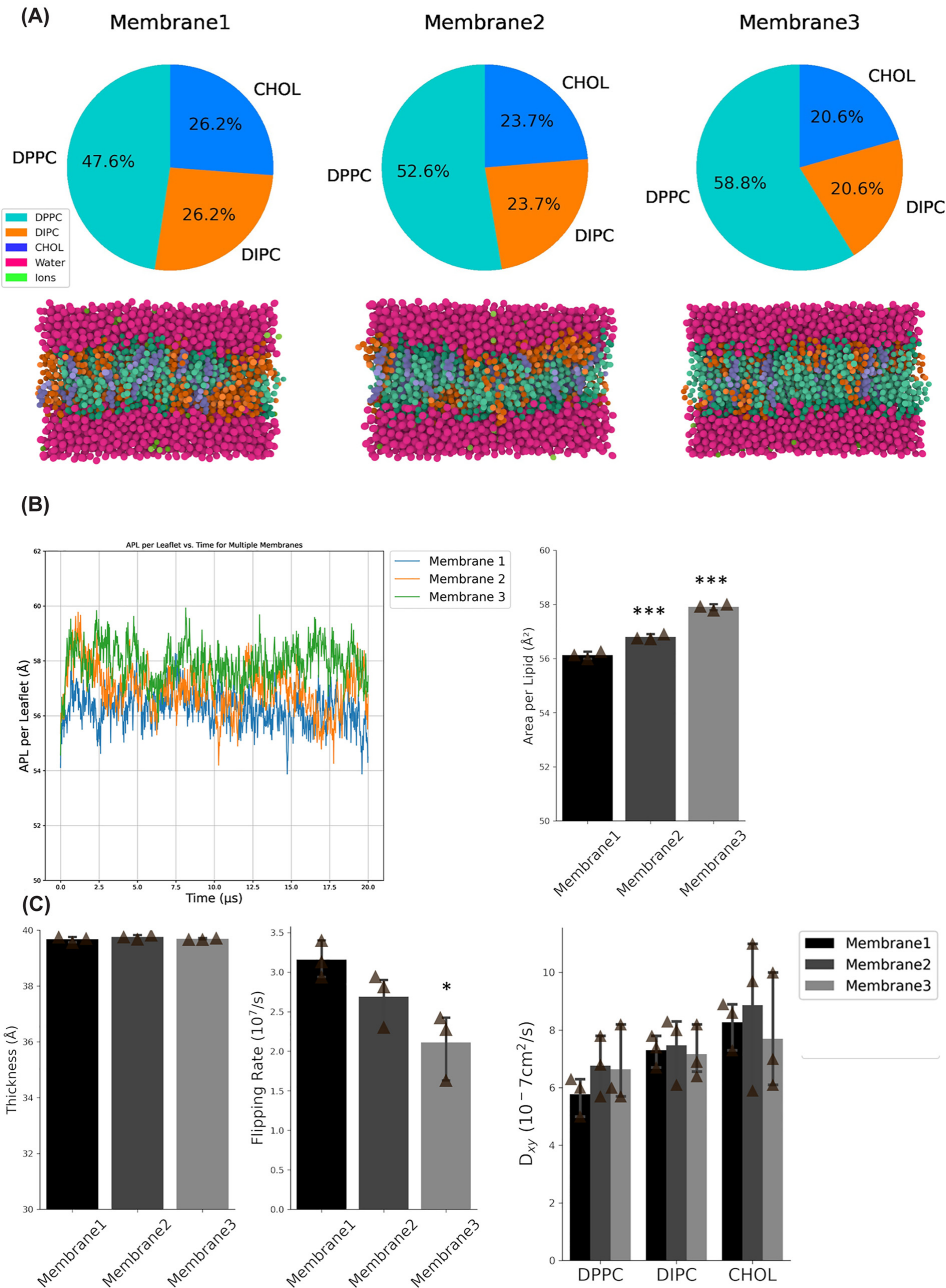
Lipid modulation affects biophysical properties of membranes (**A**) PM lipid distributions. Pie charts with the overall distribution of the lipids, as well as snapshots of the membranes of the simulations after 20 μs. (**B**) APL over 20 μs simulation and the average APL. (**C**) Membrane thickness; average CHOL flipping rate and lipid lateral self-diffusivities for each membrane. ANOVA test complemented with Tukey’s test was used for statistical analysis between groups. Data are represented as mean ± SEM. *P <0.05; ***P <0.001.

Since cholesterol levels also impact membrane physical properties, we use the MARTINI force field for coarse-grained molecular dynamics simulations to evaluate the impact of unsaturation rate and cholesterol levels on mechanical properties of the membrane by using three membranes that decrease cholesterol levels and unsaturated ratio as observed in our experimental data (Figure 5A). The membrane 1, membrane 2, and membrane 3 share a similar composition of these lipids to A172, U87MG, and T98G, respectively. APL slightly increases from membrane 1 to membrane 3, even though no changes on bilayer thickness were observed (Figure 5B). Interestingly, the lipid composition related to a more aggressive phenotype is related to a decreased cholesterol flip-flop rate (Figure 5C). Changes in lipid composition observed on glioma cells impact the membrane’s mechanical property and cholesterol trafficking, suggesting that biophysical properties of the membrane are modulated according to aggressiveness.

### Sphingomyelin to sphingosine-1-phosphate conversion dictates glioma proliferation and prognosis

**Figure 6:**
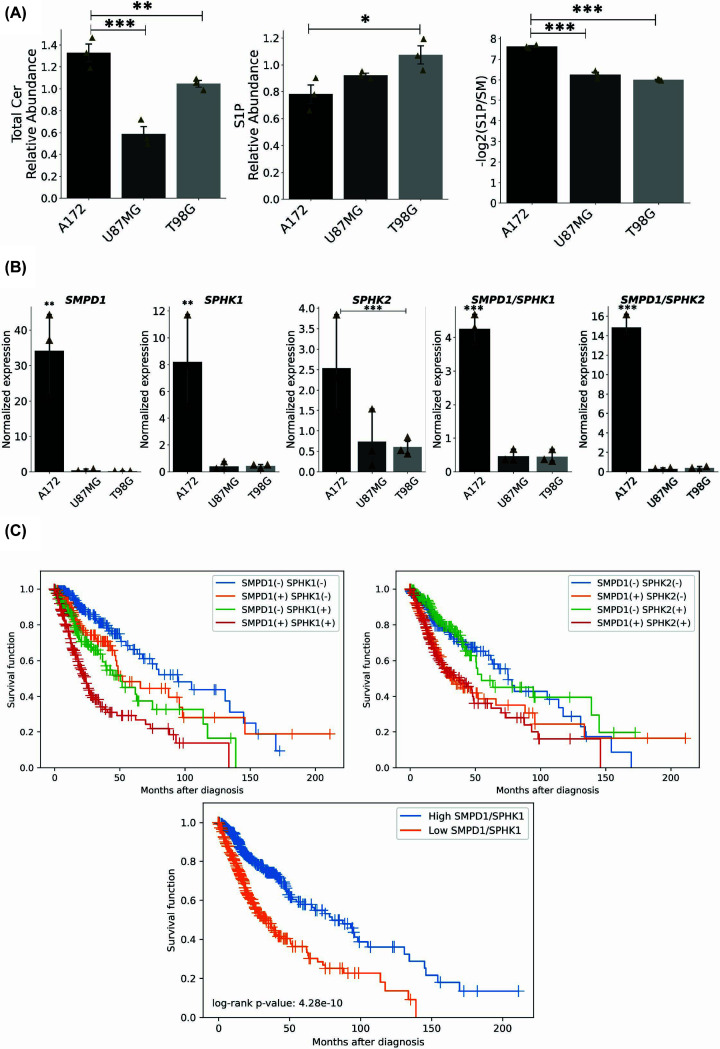
Impact of the SM to S1P in glioma aggressiveness (**A**) Relative abundance of total ceramides and S1P, as well as the SM/S1P ratio, in each glioma cell line as determined by targeted LC-MS analysis. Data are derived from three independent biological replicates and are presented as mean ± SEM. Statistical significance between groups was assessed using one-way ANOVA followed by appropriate post hoc testing. *P <0.05; **P <0.01; ***P <0.001. (**B**) Relative expression levels of SMPD1, SPHK1, and SPHK2 normalized to HMBS in each glioma cell line, along with the calculated expression ratios SPHK1/SMPD1 and SPHK2/SMPD1. ANOVA was used for statistical comparison between groups. Data are represented as mean ± SEM. *P <0.05; **P <0.01; ***P <0.001. (**C**) Kaplan-Meier curves displaying the estimated survival probability for 4 different groups of patients according to the expressions of *SPHK* and *SMPD1* and the log rank test calculated for each comparison. For SMPD1 and SPHK1 stratification, pairwise log-rank test *P*-values were SMPD1(−) SPHK1(−) versus SMPD1(+) SPHK1(−): *P* = 0.029; SMPD1(−) SPHK1(−) versus SMPD1(−) SPHK1(+): *P* = 5.598 × 10^−5^; SMPD1(−) SPHK1(−) versus SMPD1(+) SPHK1(+): *P* = 9.806 × 10^−16^; SMPD1(+) SPHK1(−) versus SMPD1(−) SPHK1(+): *P* = 0.160; SMPD1(+) SPHK1(−) versus SMPD1(+) SPHK1(+): *P* = 1.838 × 10^−6^; SMPD1(−) SPHK1(+) versus SMPD1(+) SPHK1(+): *P* = 0.0004; For SMPD1 and SPHK2 stratification, pairwise log-rank test *P*-values were SMPD1(−) SPHK2(−) versus SMPD1(+) SPHK2(−): *P* = 0.0025; SMPD1(−) SPHK2(−) versus SMPD1(−) SPHK2(+): *P* = 0.57; SMPD1(−) SPHK2(−) versus SMPD1(+) SPHK2(+): *P* = 0.0001; SMPD1(+) SPHK2(−) versus SMPD1(−) SPHK2(+): *P* = 0.0002; SMPD1(+) SPHK2(−) versus SMPD1(+) SPHK2(+): *P* = 0.69; SMPD1(−) SPHK2(+) versus SMPD1(+) SPHK2(+): *P* = 4.994 × 10^−5^.

Since the patient data showed that the sphingolipid pathway could be modulated in malignancy, we performed a 2D NMR HSQC analysis to evaluate these lipids (Supplementary Figure S7). Furthermore, we complement sphingolipid quantification with targeted LC-MS lipidomic analysis. The LC-MS results confirmed modulation of the sphingolipid pathway and demonstrated that more aggressive cell lines presented higher levels of S1P together with relatively lower levels of total ceramides and sphingomyelins (Figure 6). Moreover, ratio analyses revealed higher Cer/SM conversion, increased S1P/sphingosine conversion, and enhanced overall SM-to-S1P flux in aggressive cells, supporting increased pathway activity toward S1P synthesis (Figure 6A). Given the lipidomic evidence indicating differential remodeling of the SM-ceramide-S1P axis between less aggressive and aggressive glioma cells, we next examined whether transcriptional regulation of key enzymes in this pathway paralleled the metabolic changes observed by LC-MS. Specifically, we evaluated the expression of SMPD1, which catalyzes sphingomyelin hydrolysis to ceramide, and SPHK1/2, which convert sphingosine to S1P. As shown in Figure 6A, the A172 cell line presented a higher expression of both SMPD1 and SPHK1 compared with the more aggressive cell lines. However, when considering the balance between ceramide generation and S1P synthesis, the SMPD1/SPHK1 ratio was markedly higher in the less proliferative cells. This transcriptional ratio mirrors our LC-MS findings, in which less aggressive cells accumulated higher levels of total ceramides, whereas aggressive cells displayed features consistent with enhanced downstream utilization of ceramide and reduced steady-state ceramide abundance.

Consistent with this interpretation, analysis of patient datasets revealed that higher-grade gliomas exhibit increased expression of both SMPD1 and SPHK1 (Figure 3). Kaplan–Meier analysis has shown that higher expression of SMPD1 and SPHK1 highly decreases the patient's survival, whereas there are not very significant differences when only one gene is more highly expressed (Figure 6B). The same is not observed with SMPD1 and SPHK2, where only a higher expression of SPHK2 is related to decreased survival, meaning that the SMPD1/SPHK1 and not the SMPD1/SPHK2 axis is related to aggressiveness in patients. Furthermore, a higher SMPD1/SPHK1 ratio was related to better overall survival, suggesting that SM to S1P via the SMPD1/SPHK1 axis would be related to aggressiveness and patient prognosis.

In addition, untargeted LC-MS lipidomic analysis revealed broader lipid remodeling associated with aggressiveness (Supplementary Figures S8 and S9). The less proliferative A172 cells showed enrichment of lipid storage-related pathways, including increased triacylglycerols (TGs) and higher representation of polyunsaturated fatty acid (PUFA)-containing species, suggesting a greater allocation toward lipid reserve pools. In contrast, the more proliferative cell lines exhibited enrichment of glycerophospholipid-associated pathways, particularly PC and phosphatidylethanolamine (PE) species, consistent with enhanced membrane biosynthesis demand capacity (Supplementary Figures S8 and S10). These findings complement and extend the NMR data, which indicated differential membrane lipid composition between the cell lines, further supporting coordinated lipid remodeling associated with proliferative capacity. Targeted sphingolipid profiling further demonstrated marked remodeling of ceramide and sphingomyelin species composition (Supplementary Figures S10 and S11). Less aggressive cells displayed higher levels of long-chain ceramides, particularly C16:0 and C18:0 species, whereas more aggressive cells exhibited an increased very-long-chain-to-long-chain ceramide ratio, characterized by enrichment of C24:1 ceramide (Supplementary Figure S11). Consistent with these findings, analysis of patient transcriptomic data revealed elevated expression of LASS2 (CERS2) in GBM samples. As CERS2 preferentially synthesizes very-long-chain ceramides, including C24 species, its increased expression supports the enrichment of C24:1 ceramide observed in aggressive cells and further suggests coordinated regulation of ceramide chain-length composition in glioma progression.

Differences were also observed in glycosphingolipids, where monohexosylceramides (Hex1Cer) were enriched in less aggressive cells, whereas dihexosylceramides (Hex2Cer) were higher in aggressive cells. Supporting this observation, analysis of patient transcriptomic data revealed that expression of *B4GALT5*, which catalyzes the galactosylation of Hex1Cer to generate Hex2Cer (lactosylceramide), was elevated in GBM samples (Supplementary Figure S6). Furthermore, additional key genes involved in glycosphingolipid metabolism were differentially expressed according to glioma grade (Supplementary Figure S6), indicating coordinated modulation of this pathway. These findings suggest that glycosphingolipid remodeling—potentially driven by enhanced Hex1Cer-to-Hex2Cer conversion—may represent an additional metabolic adaptation associated with glioma progression.

Taken together, our findings indicate that glioblastoma aggressiveness is associated not only with altered expression of key enzymes within the SM-ceramide-S1P axis but also with coordinated lipid remodeling at the metabolite level. While elevated SMPD1 expression correlates with higher glioma grade, its biological impact appears to depend on its balance with SPHK1, as reflected by the SMPD1/SPHK1 ratio, which aligned with both cellular proliferative capacity and patient survival trends. Consistent with this transcriptional regulation, lipidomic analyses revealed reduced total ceramide levels in more aggressive cells; selective enrichment of very-long-chain ceramides—particularly C24:1—alongside a higher very-long-chain-to-long-chain ceramide ratio; and remodeling of glycosphingolipids characterized by increased Hex2Cer relative to Hex1Cer. These metabolic shifts suggest that aggressive glioma cells reprogram sphingolipid metabolism through both altered enzyme expression and ceramide species redistribution, potentially favoring survival-associated membrane adaptation and signaling. However, given the complexity of sphingolipid networks and the limitations of *in vitro* models and bulk transcriptomic datasets, further mechanistic and subtype-specific studies are warranted to clarify the causal role of this metabolic axis in glioma progression.

## Discussion

We could identify 29 lipid-related genes that are correlated to prognosis in patient data, and from the risk-signature construction based on these genes expression, we showed a significant difference in overall survival according to the risk. ECM-related genes were positively correlated to the risk score, while the gene expression data suggested that structural and signaling lipid pathways could be involved in glioma aggressiveness. GBM patients displayed elevated expression of genes crucial for synthesizing phosphatidylcholine and phosphoethanolamine, which are essential components of the plasma membrane [38]. Taken all together, the data indicates that a cross-talk between membrane composition and its role in regulating the ECM would play an important role in the aggressiveness phenotype. Mechanical and physical properties of ECM control cell proliferation, and it was shown that it also regulates lipid metabolism through different pathways [39,40]. Phosphoethanolamine plays an important role promoting membrane protein folding [41]; interestingly, ER lipid-associated pathways are also up-regulated in the GBM patients, suggesting that high levels of PE could be due to a response to ER stress. It has been shown that GBM highly expresses unfold protein response markers; therefore, high levels of PE could sustain oncogenic ER stress and promote glioblastoma survival [42]. Accumulation of choline-containing phospholipids has already been described in several cancers, and its metabolism plays an important pro-tumoral role supporting proliferative phenotype [9]. Here, we demonstrated in glioblastoma cell lineages with different tumorigenic phenotypes (A172, U87MG, and T98G) [36,37] that levels of choline-containing lipids increase according to the aggressiveness of GBM cells, confirming what the expression data analysis suggests. Higher expression of genes involved in the last step of phosphatidylcholine synthesis, along with lower expression of genes involved in phosphatidylcholine degradation in GBM patients, could be related to higher expression of this lipid, therefore being an important pathway to glioma aggressiveness maintenance. Moreover, according to the NMR profile and LC-MS lipidomic data, the analyzed cell lines present different levels of choline, phosphocholine, and phosphatidylcholine. The different composition observed in cell lines could also suggest that they would be related to glioma proliferation and aggressive phenotype.

PTEN mutation is strongly related to worse prognosis and malignancy in glioma [43]. Interestingly, we have noticed that lower expressions of several phosphatidylinositol-related genes are correlated to higher-grade gliomas. Since PTEN mutation is more common in higher-grade gliomas, we evaluated whether the PI pathway modulation would be due to a higher PTEN mutation prevalence. PI pathway-associated genes are down-regulated in PTEN-mutated patients regardless of the glioma grade, which could be explained by a negative feedback regulation of gene expression. Cholesterol metabolism was negatively associated with PTEN mutation. Since PTEN mutation is related to worse prognosis and more aggressive types of gliomas [43], it would reinforce that cholesterol metabolism is more related to less aggressive types of gliomas. Here, we showed that several genes important to cholesterol biosynthesis are down-regulated in GBM. Furthermore, important enzymes that regulate cholesterol biosynthesis, HMGCR and SQLE, are related to higher survival rates. We have determined the cholesterol profile in our cellular model, in which cholesterol decrease is related to a higher proliferation rate in PTEN-mutant glioblastoma cells.

The MARTINI molecular dynamics simulations show that a lower unsaturation rate and lower levels of cholesterol, similar to more proliferative glioblastoma cells, decrease cholesterol flipping rate and increase APL, suggesting that physical membrane properties changes could be related to higher malignancy. APL is a measure of membrane packing and is closely related to the phase and fluidity of the membrane. It has been described that decreased levels of cholesterol in the membrane in cancer cells lead to an increase in APL, which could be explained by a lower ordering of the lipid chains [44–46]. Furthermore, cholesterol flip-flop rate could be modulated by its own levels and unsaturation rate [47,48]. Here, we showed that the lipid composition of more aggressive cells leads to a more fluid membrane and changed cholesterol trans-bilayer distribution that would impact the organization of the cell membrane. More fluid membranes were already described in cancer cells and promote a more aggressive phenotype by facilitating tissue invasion and metastasis [38,49]. Furthermore, the cholesterol efflux receptor was related to controlling the membrane fluidity in cancer, and by decreasing that fluidity, the cell motility and the epithelial-mesenchymal transition can be reduced as well as metastasis inhibition [50].

It was described that increasing PUFA regulates domains of the membrane by its low affinity to cholesterol, which would modulate protein signaling [51]; therefore, the levels of PUFA and cholesterol could not only be modulated to regulate malignant membrane biophysical properties but also tumoral signaling pathways in more proliferative glioblastoma cells.

Cholesterol intracellular trafficking is tightly correlated to sphingomyelin levels by acid SMase activity [52–55]. Here, we showed that a less aggressive glioblastoma cell line presents higher levels of cholesterol and lower levels of sphingomyelin, suggesting a dual role of those lipids in aggressiveness.

In addition, the untargeted LC-MS lipidomic analysis further revealed differences in broader lipid pathway enrichment between the cell lines. The less proliferative A172 cells showed enrichment of lipid storage-related pathways, including increased TGs and higher representation of PUFA-containing species, suggesting a greater allocation toward lipid reserve pools. In contrast, the more proliferative cell lines exhibited enrichment of glycerophospholipid-associated pathways, particularly PC and PE species, consistent with enhanced membrane biosynthesis demands. These findings complement and extend the NMR data, which indicated differential membrane lipid composition between the cell lines, further supporting coordinated lipid remodeling associated with proliferative capacity.

The less proliferative glioblastoma cell (A172) shows higher expression of acid sphingomyelinase *SMPD1*, suggesting that this cell lineage has higher conversion of sphingomyelin to ceramide. Consistent with this transcriptional profile, targeted LC-MS lipidomic analysis demonstrated that the less proliferative cells exhibit higher total ceramide levels, particularly long-chain species such as C16:0 and C18:0. In contrast, the more aggressive cells showed reduced total ceramide levels but an increased very-long-chain-to-long-chain ceramide ratio, characterized by enrichment of C24:1 ceramide, indicating selective ceramide species remodeling rather than uniform depletion. On the other hand, S1P was shown to promote pro-tumoral effects, being correlated to glioma malignancy [15]. According to that, we showed from TCGA data that sphingolipid-related genes are up-regulated in higher-grade glioma, and it suggests that S1P would be more present in this more aggressive type. Furthermore, since ceramide formed from sphingomyelin could be further converted to S1P, we analyzed the SMPD1 to SPHKs ratios (SMPD1/SPHK1 and SMPD1/SPHK2) in the glioblastoma cells. The less proliferative cell line showed higher SMPD1/SPHK1 and SMPD1/SPHK2 ratios, suggesting more conversion of sphingomyelin to ceramide, whereas the more proliferative cells presented ratios lower than 1 suggesting more conversion to S1P. In agreement with this metabolic shift, LC-MS analysis also revealed remodeling of glycosphingolipids, with enrichment of Hex2Cer relative to Hex1Cer in more aggressive cells, supporting enhanced downstream sphingolipid utilization. Finally, higher expression of both genes SMPD1 and SPHK1 drastically decreases the overall survival rate of glioma patients. Taken all together, the conversion of sphingomyelin to S1P has shown to be important for glioma growth and aggressiveness. These findings suggest at potential link between SMPD1/SPHK1 expression and glioblastoma aggressiveness; however, further research is necessary to investigate the levels of S1P and validate the hypotheses that SM to S1P conversion is linked to aggressiveness.

## Conclusions

The present study investigated the lipid metabolism in GBM aggressiveness, highlighting the potential for therapeutic and diagnostics. By integrating transcriptomic data from TCGA and lipidomic profiles of GBM cell lines, we identified 29 lipid-related genes linked to prognosis and established a risk signature correlated with extracellular matrix components. Our findings reveal that GBM aggressiveness is associated with distinct alterations in membrane composition, including increased phospholipid content, reduced cholesterol levels, and decreased fatty acid unsaturation, which collectively impact membrane properties. The sphingomyelinase-S1P axis, involving SMPD1 and SPHK1, emerged as a key pathway in glioma progression. These insights provide a foundation for further exploration of lipid-related pathways as potential biomarkers and therapeutic targets in GBM.

The present study has limitations that should be considered when interpreting the findings. First, although the integration of transcriptomic, targeted, and untargeted lipidomic data; survival analyses; and biophysical modeling provides multi-level evidence linking sphingolipid remodeling to glioblastoma aggressiveness, the data primarily demonstrate associations rather than direct causality. Further functional perturbation experiments targeting key components of the SMPD1/SPHK axis will be necessary to establish a definitive mechanistic role. Our *in vitro* models represent PTEN-mutant, IDH-wild-type GBM, and while stratified analyses confirmed that the observed associations persist within IDH-wild-type patients, the limited availability of stable IDH-mutant low-grade glioma cellular models restricts direct experimental comparison across molecular subtypes. Future studies incorporating functional manipulation, subtype-specific models, and expanded proteomic validation will be essential to refine and extend the mechanistic framework proposed here.

## Highlights

- NMR lipidomic data identifies aggressiveness markers that can be used for non-invasive MRS.
- Glioma aggressiveness linked to lipid composition.
- Membrane alterations correlate with glioma aggression.
- Sphingolipid signaling crucial in glioma.

## Supplementary Material

Supplementary Figures S1-S12 and Tables S1-S2

## Data Availability

The authors confirm that the data supporting the findings of the present study are available within the article and its supplementary materials.

## Open Access

Open access for this article was enabled by the participation of University of Groningen in an all-inclusive Read & Publish agreement with Portland Press and the Biochemical Society.

## Abbreviations

APL: area per lipid
CDP: cytidine diphosphate
Cer: ceramide
CV: coefficient of variation
DE: differently expressed
DMEM: Dulbecco MEM media
DMSO: dimethyl sulfoxide
ECM: extracellular matrix
EDTA: ethylenediaminetetraacetic acid
ER: endoplasmic reticulum
GBM: Glioblastoma
GO: Gene Ontology
HSQC: heteronuclear single quantum coherence spectroscopy
LC- MS: liquid chromatography-mass spectrometry
LGG: low grade glioma
MSM: methylsulfonylmethane
MRS: Magnetic Resonance Spectroscopy
NMR: nuclear resonance magnetic
PC: phosphatidylcholine
PCR: polymerase chain reaction
PI: phosphatidylinositol
PTEN: phosphatase and tensin homolog
PTENmut: PTEN mutant
PUFA: polyunsaturated fatty acid
S1P: sphingosine-1-phosphate
SM: sphingomyelin
SMPD: sphingomyelin phosphodiesterase
SPHK: sphingosine kinase
SQLE: squalene epoxidase
TCGA: The Cancer Genome Atlas
TSP: trimethylsilylpropanoic acid
QC: quality control
