# Supplementary material for: Lipid metabolism as a marker for glioma aggressiveness
Source: Biosci Rep. 2026 Jun 3;46(6):BSR20253270. doi: 10.1042/BSR20253270 (PMC13234697; doi:10.1042/BSR20253270)
Supplement: Supplementary Figures S1-S12 and Tables S1-S2 [file BSR-2025-3270_supp.pdf]

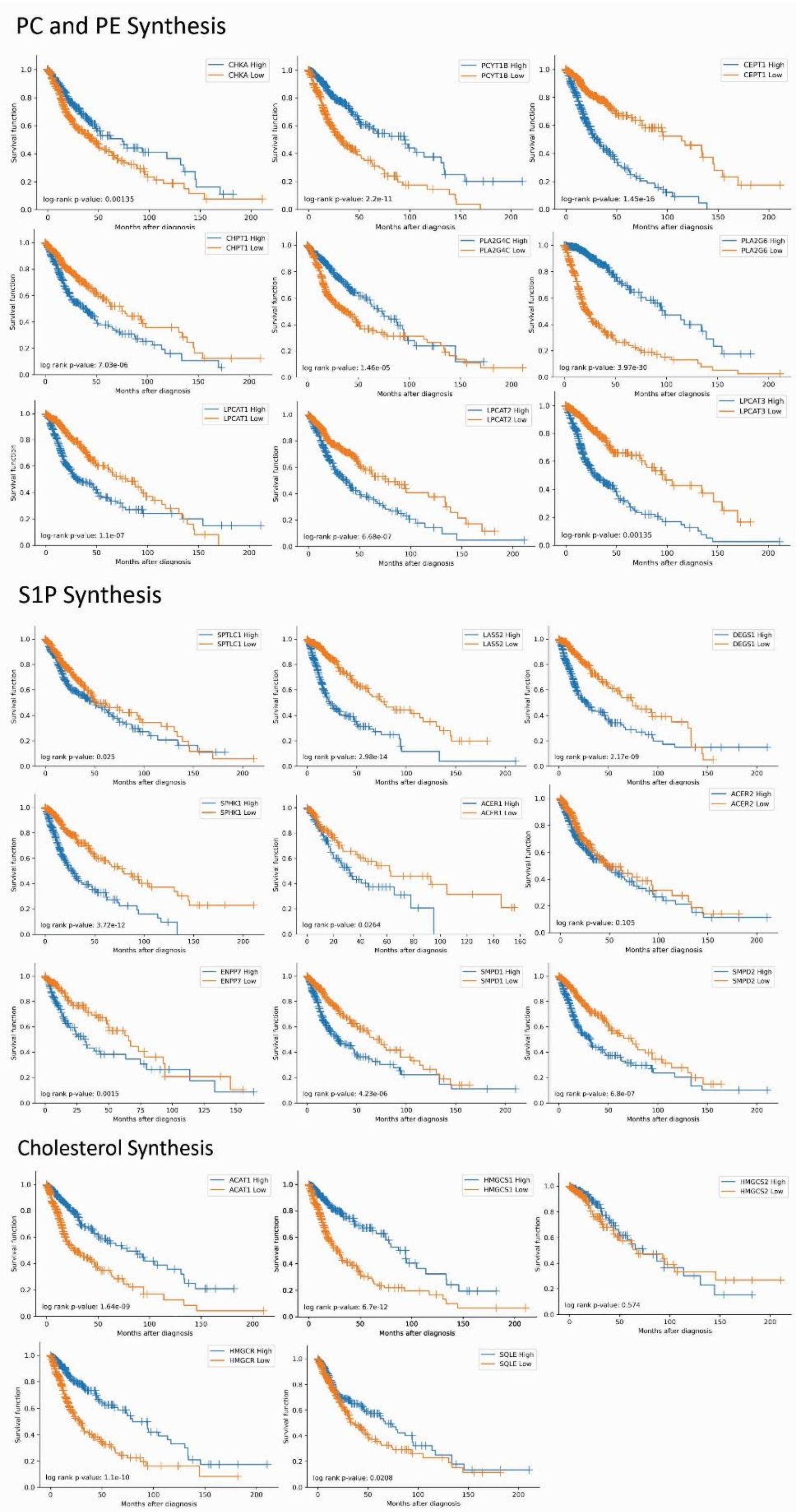

FigS1. **Lipid related genes linked to glioma survival**. Survival curves of genes important for metabolism are represented, the median value of each gene expression was used as cutoff (n=681).

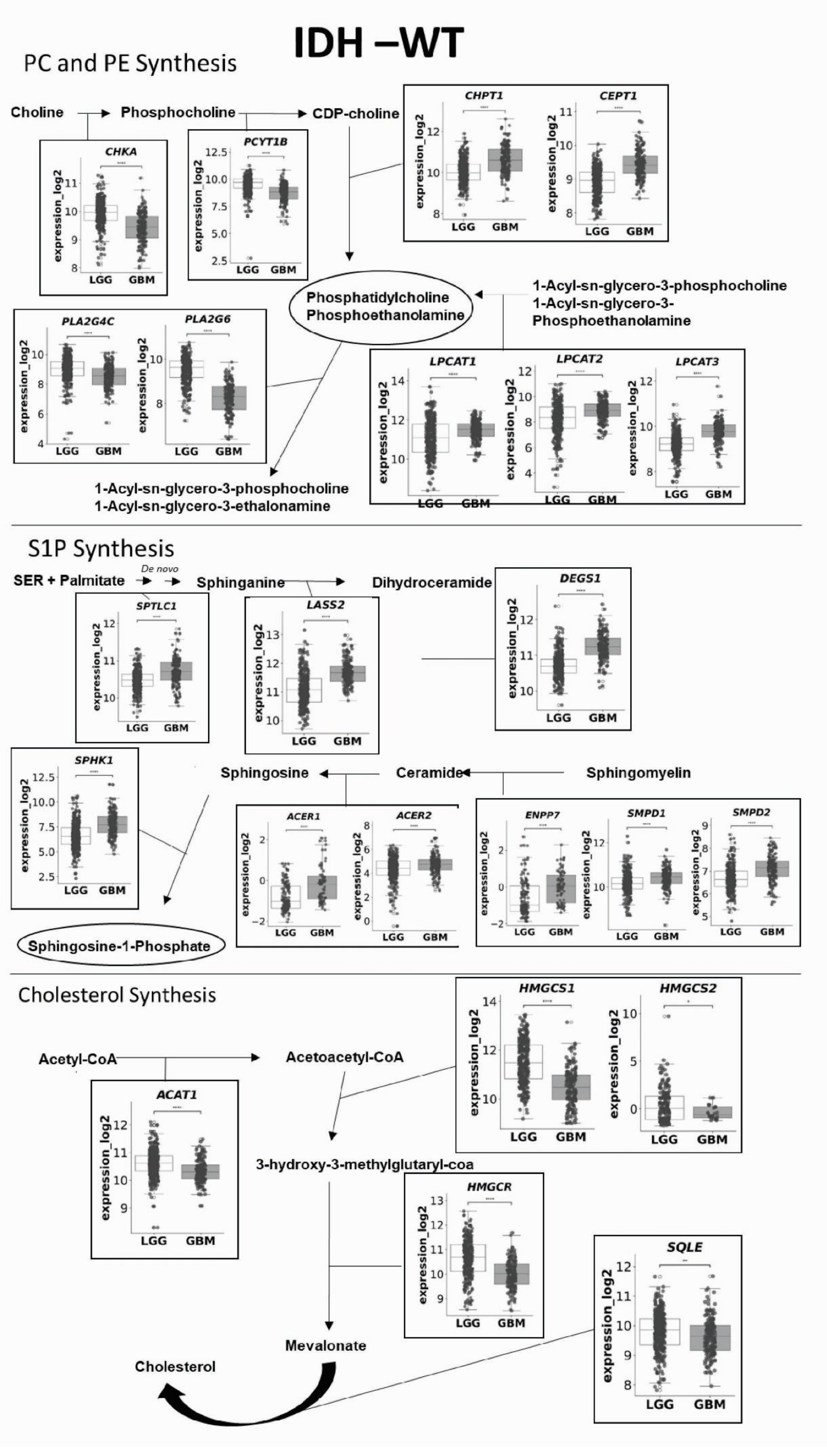
IDH –WT
PC and PE Synthesis
Choline
Phosphocholine
CDP-choline
CHKA
PCYT1B
CHPT1
CEPT1
Phosphatidylcholine
Phosphoethanolamine
1-Acyl-sn-glycero-3-phosphocholine
1-Acyl-sn-glycero-3-
Phosphoethanolamine
PLA2G4C
PLA2G6
LPCAT1
LPCAT2
LPCAT3
1-Acyl-sn-glycero-3-phosphocholine
1-Acyl-sn-glycero-3-ethalonamine
S1P Synthesis
SER + Palmitate
De novo
Sphinganine
Dihydroceramide
SPTLC1
LASS2
DEGS1
SPHK1
Sphingosine
Ceramide
Sphingomyelin
ACER1
ACER2
ENPP7
SMPD1
SMPD2
Sphingosine-1-Phosphate
Cholesterol Synthesis
Acetyl-CoA
Acetoacetyl-CoA
HMGCS1
HMGCS2
ACAT1
3-hydroxy-3-methylglutaryl-coa
HMGCR
SQLE
Mevalonate
Cholesterol
expression_log2
LGG
GBM

FigS2. Lipid metabolism pathways related to glioma aggressiveness in ID-wt. Representation of the three most important lipid metabolism pathways with the expressions of the genes that are being modulated according to aggressiveness in IDH-wt patients, T-test was used to compare between groups *p<0,05; **p<0,01;***p<0,001 (n=232).

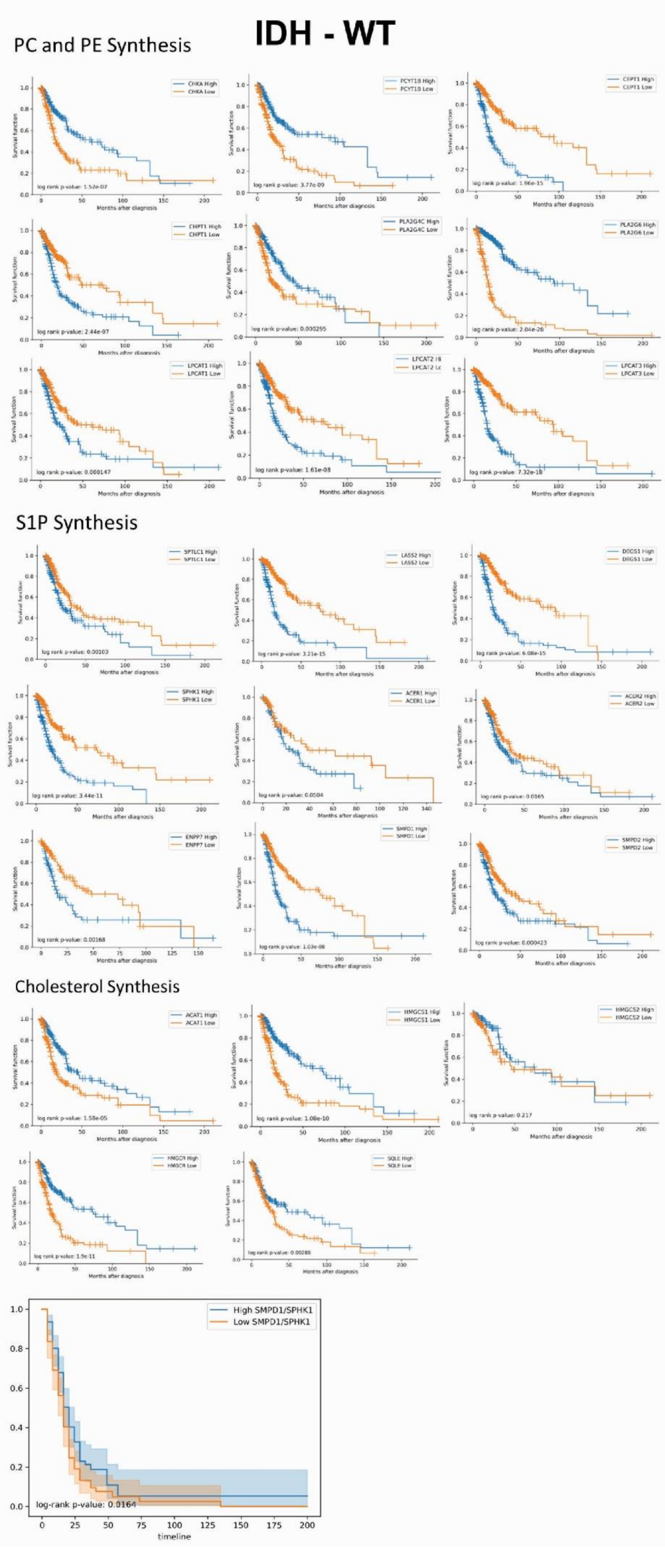
IDH - WT
PC and PE Synthesis
S1P Synthesis
Cholesterol Synthesis
High SMPD1/SPHK1
Low SMPD1/SPHK1
log-rank p-value: 0.0164
timeline

FigS3. **Lipid related genes linked to glioma survival in IDH-wt**. Survival curves of genes important for metabolism in IDH-WT, are represented, the median value of each gene expression was used as cutoff (n=232).

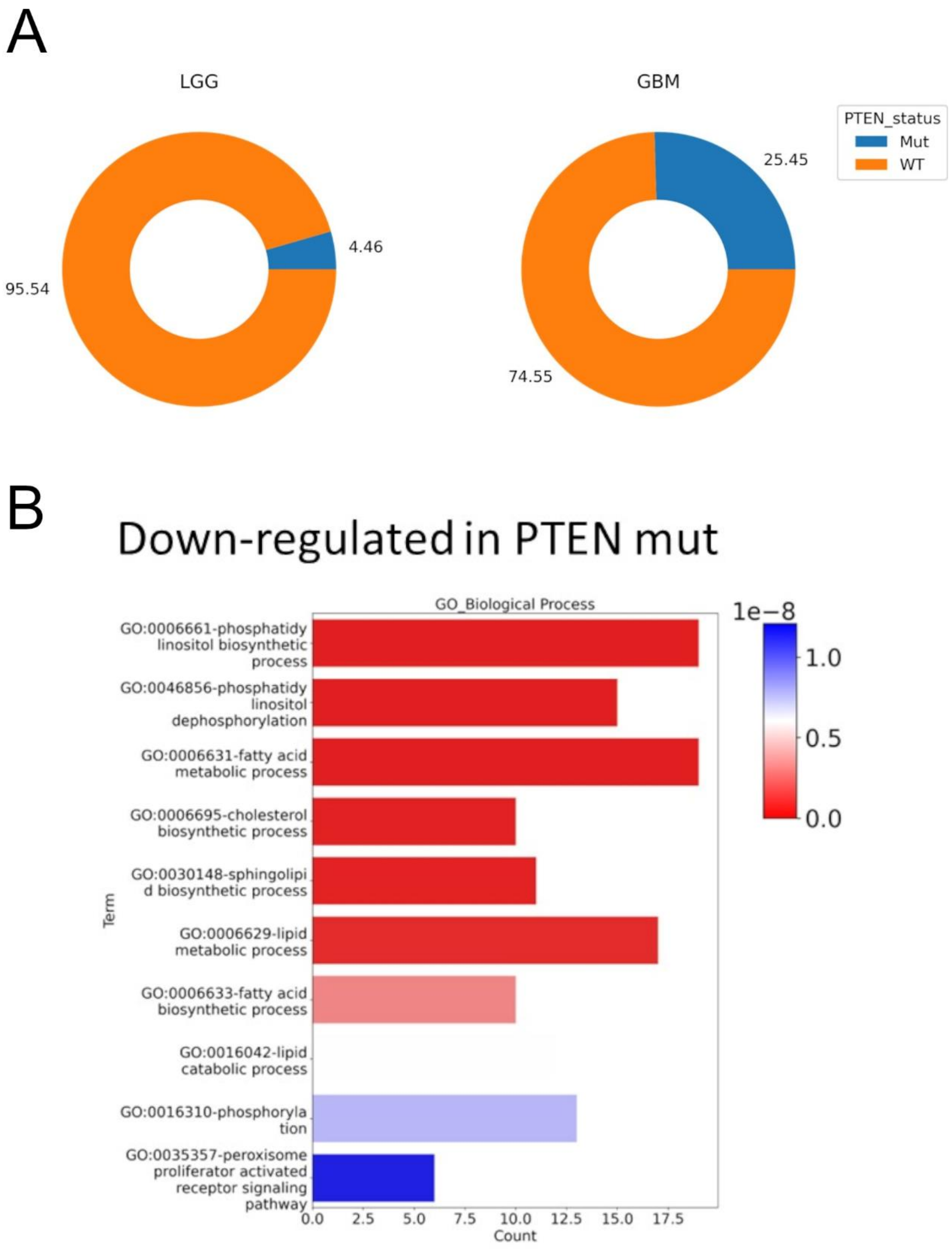

FigS4. Lipid metabolism related to PTEN mutation.

Comparing the PTEN mut vs PTEN WT population in the glioma patients and the enrichment analysis, using molecular biological process (BP) annotation from gene ontology (GO) of lipid pathways that are downregulated in the PTEN mut population. Statistical values to rank their biological significance are represented by colors.

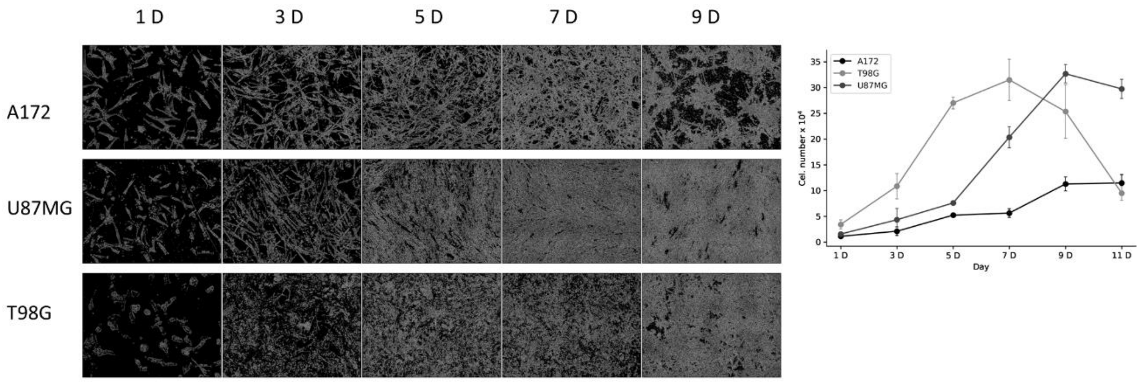

Fig S5. Glioblastoma cells with distinct aggressive phenotype. Illustrative and the proliferation curve of the three GBM cells A172, U87MG, and T98G.

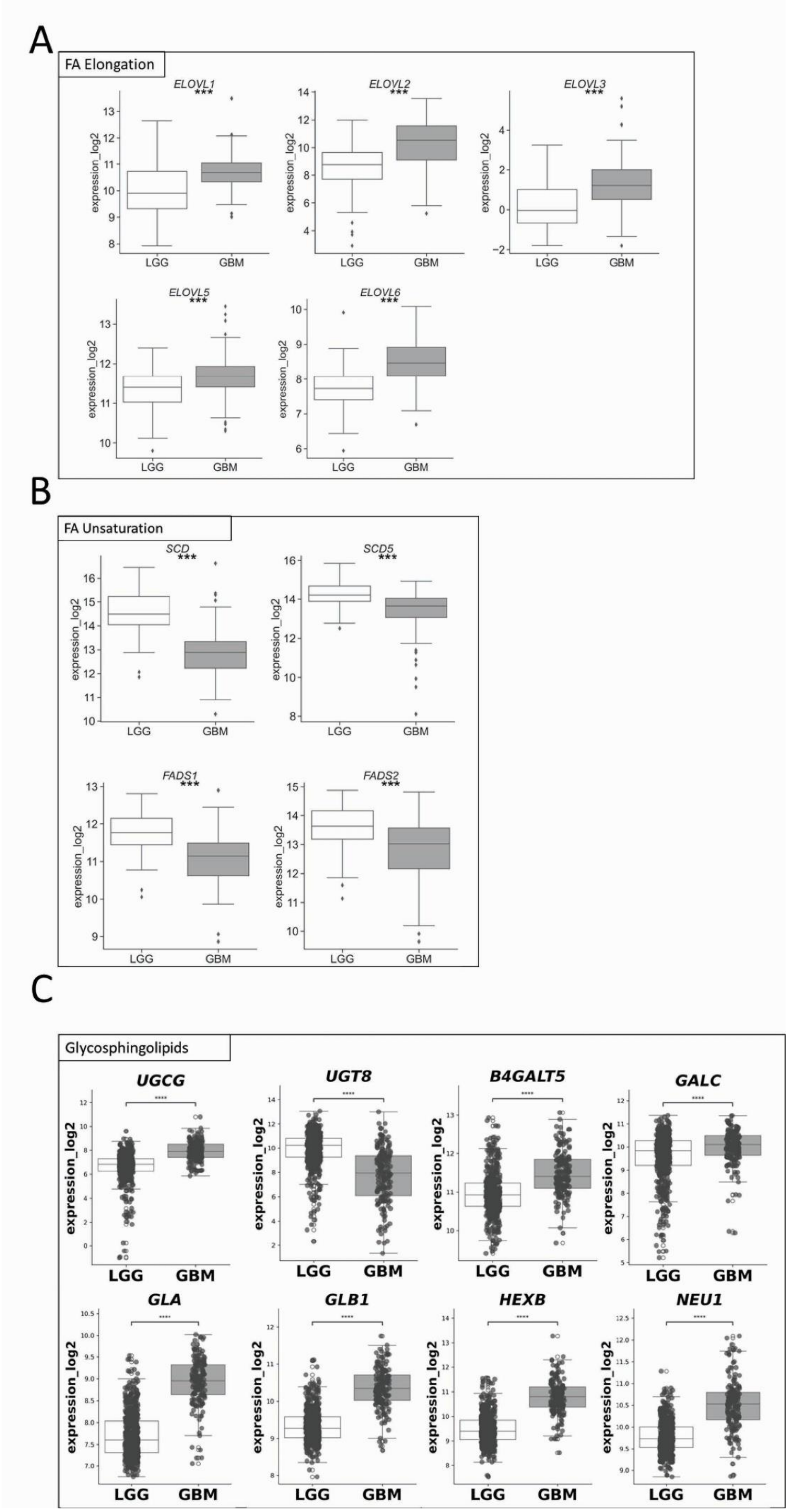
A
FA Elongation
ELOVL1
ELOVL2
ELOVL3
ELOVL5
ELOVL6
***
expression_log2
LGG
GBM
B
FA Unsaturation
SCD
SCD5
FADS1
FADS2
***
expression_log2
LGG
GBM
C
Glycosphingolipids
UGCG
UGT8
B4GALT5
GALC
GLA
GLB1
HEXB
NEU1
****
expression_log2
LGG
GBM

Fig S6. The fatty acid composition according to aggressiveness.

Comparing expression data of modulated genes important for FA elongation and unsaturation between GBM to LGG patients (n=681). T-test was used to compare between groups *p<0,05; **p<0,01;***p<0,001.

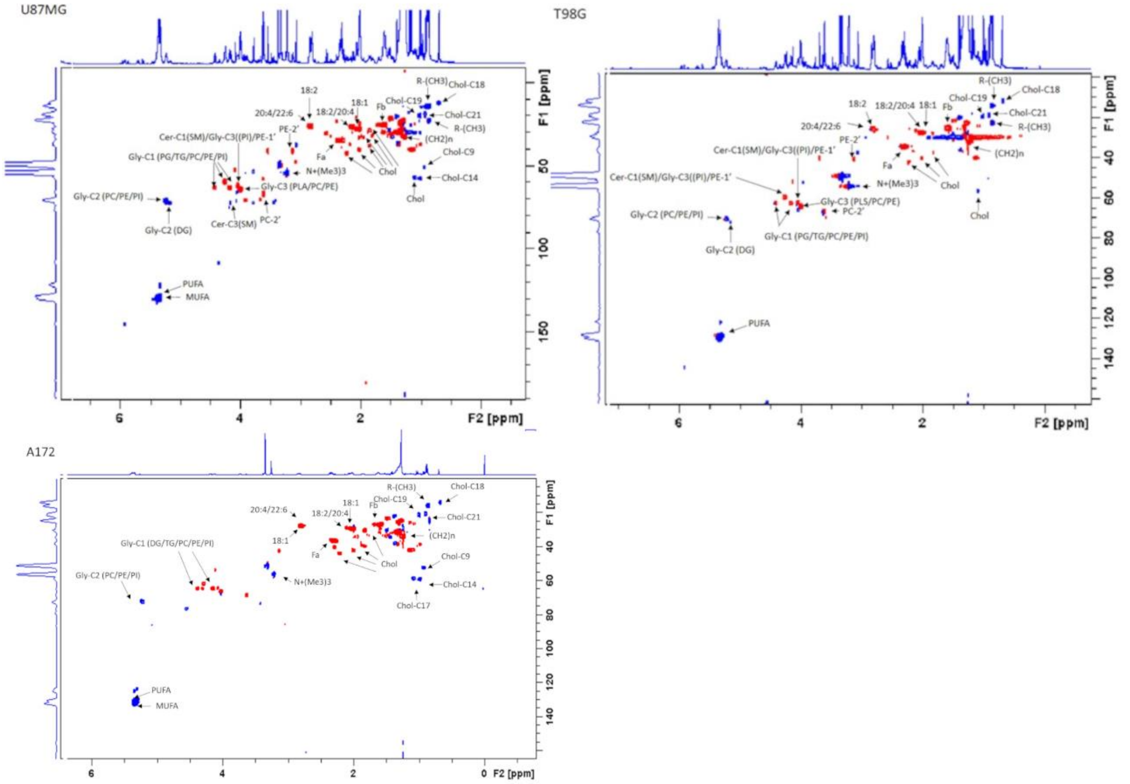

Fig S7. **Partial 2D 1H-13C multiplicity-edited HSQC NMR correlation map. 2D-NMR** of the lipid extract obtained from each glioma cell line with the principal lipid assignments. The positive phase (blue) corresponds to CH and CH3 correlations, and the negative phase (red) corresponds to CH2 correlations.

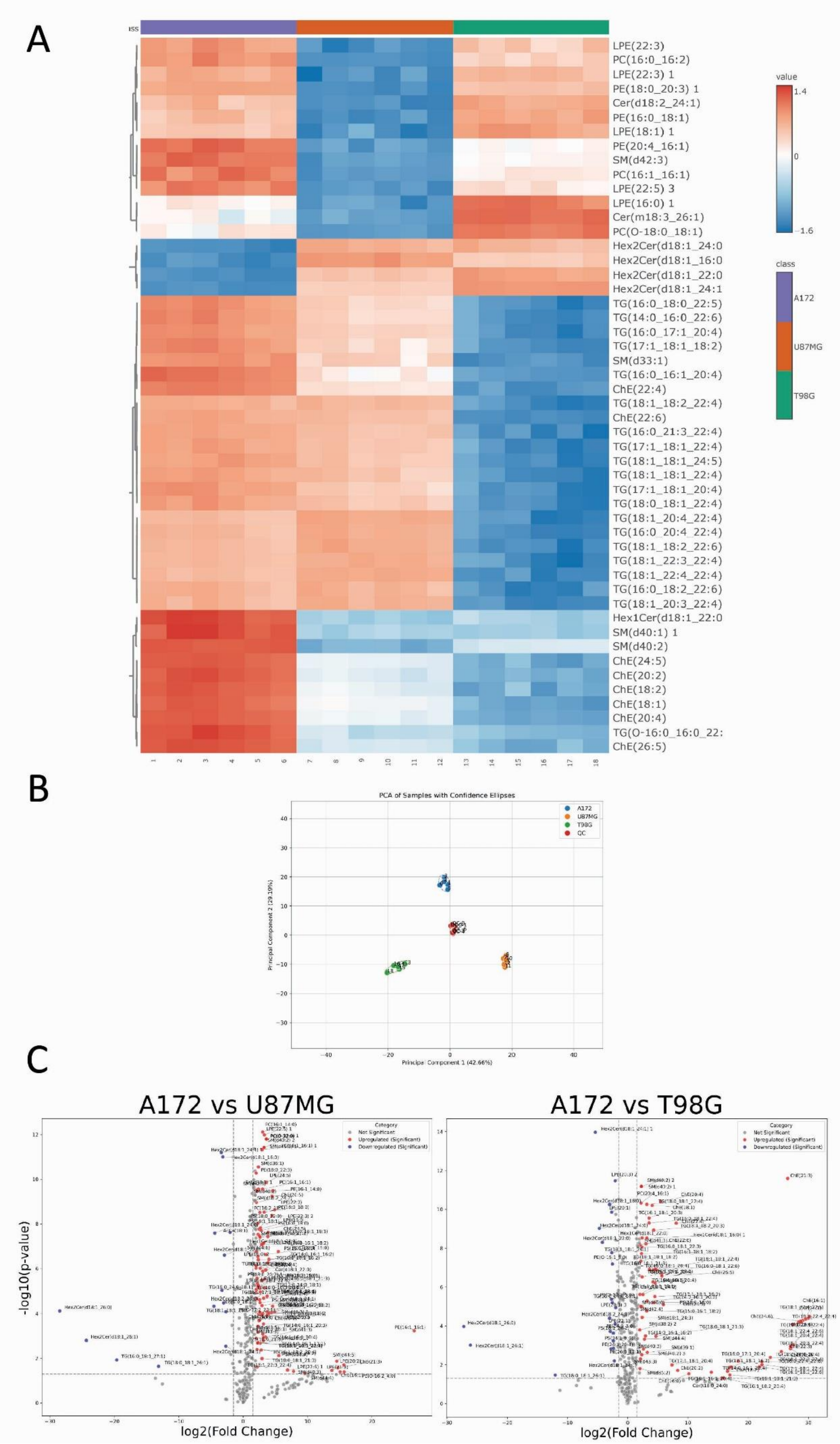

A
class
LPE(22:3)
PC(16:0_16:2)
LPE(22:3) 1
PE(18:0_20:3) 1
Cer(d18:2_24:1)
PE(16:0_18:1)
LPE(18:1) 1
PE(20:4_16:1)
SM(d42:3)
PC(16:1_16:1)
LPE(22:5) 3
LPE(16:0) 1
Cer(m18:3_26:1)
PC(O-18:0_18:1)
Hex2Cer(d18:1_24:0
Hex2Cer(d18:1_16:0
Hex2Cer(d18:1_22:0
Hex2Cer(d18:1_24:1
TG(16:0_18:0_22:5)
TG(14:0_16:0_22:6)
TG(16:0_17:1_20:4)
TG(17:1_18:1_18:2)
SM(d33:1)
TG(16:0_16:1_20:4)
ChE(22:4)
TG(18:1_18:2_22:4)
ChE(22:6)
TG(16:0_21:3_22:4)
TG(17:1_18:1_22:4)
TG(18:1_18:1_24:5)
TG(18:1_18:1_22:4)
TG(17:1_18:1_20:4)
TG(18:0_18:1_22:4)
TG(18:1_20:4_22:4)
TG(16:0_20:4_22:4)
TG(18:1_18:2_22:6)
TG(18:1_22:3_22:4)
TG(18:1_22:4_22:4)
TG(16:0_18:2_22:6)
TG(18:1_20:3_22:4)
Hex1Cer(d18:1_22:0
SM(d40:1) 1
SM(d40:2)
ChE(24:5)
ChE(20:2)
ChE(18:2)
ChE(18:1)
ChE(20:4)
TG(O-16:0_16:0_22:
ChE(26:5)
1 2 3 4 5 6 7 8 9 10 11 12 13 14 15 16 17 18
value
1.4
0
−1.6
class
A172
U87MG
T98G
B
PCA of Samples with Confidence Ellipses
A172
U87MG
T98G
QC
Principal Component 1 (42.66%)
Principal Component 2 (29.10%)
C
A172 vs U87MG
A172 vs T98G
Category
Not Significant
Upregulated (Significant)
Downregulated (Significant)
log2(Fold Change)
-log10(p-value)

Fig S8. Global lipidomic profiling distinguishes less and more aggressive glioblastoma cell lines.

(A) Heatmap representation of normalized lipid abundances across A172, T98G, and U87MG cell lines derived from one biological replicate representative of three independent biological experiments per cell line ($n = 6$ replicates per group). Lipid species were scaled and centered prior to clustering. The most differential lipid are highlighted

(B) Volcano plot comparing A172 versus T98G cells. Each point represents an individual lipid species. The x-axis indicates log2 fold change, and the y-axis shows –log10 (adjusted p-value). Lipids meeting the significance threshold (adjusted $p < 0.05$ and $|\log2FC| \geq 1$) are highlighted, illustrating lipid classes enriched in either less or more aggressive phenotypes.

(C) Volcano plot comparing A172 versus U87MG cells. Differentially abundant lipid species were identified using the same statistical criteria as in panel (B).

(D) Principal component analysis (PCA) of untargeted lipidomic data. Each point represents a biological replicate. PCA was performed on normalized lipid abundance values. The first two principal components (PC1 and PC2) explain the indicated percentage of total variance. Clear separation of A172 from T98G and U87MG along PC1 reflects distinct global lipidomic signatures associated with proliferative capacity.

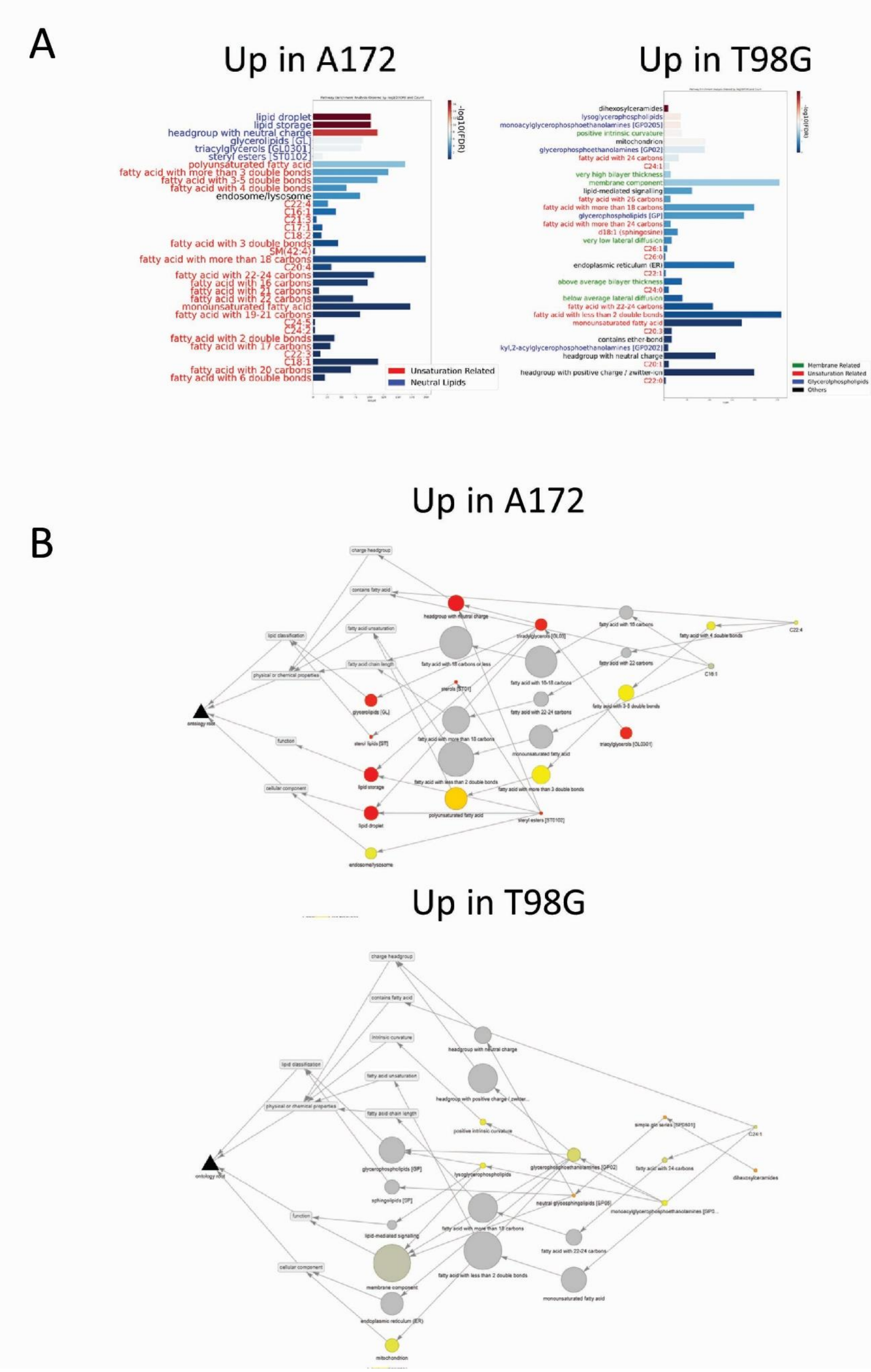
A
Up in A172
Up in T98G
lipid droplet
lipid storage
headgroup with neutral charge
glycerolipids [GL]
triacylglycerols [GL0301]
steryl esters [ST0102]
polyunsaturated fatty acid
fatty acid with more than 3 double bonds
fatty acid with 3-5 double bonds
fatty acid with 4 double bonds
endosome/lysosome
C22:4
C16:1
C21:3
C17:1
C18:2
fatty acid with 3 double bonds
SM(42:4)
fatty acid with more than 18 carbons
C20:4
fatty acid with 22-24 carbons
fatty acid with 16 carbons
fatty acid with 21 carbons
fatty acid with 22 carbons
monounsaturated fatty acid
fatty acid with 19-21 carbons
C24:5
C24:2
fatty acid with 2 double bonds
fatty acid with 17 carbons
C22:3
C18:1
fatty acid with 20 carbons
fatty acid with 6 double bonds
-log10(FDR)
Unsaturation Related
Neutral Lipids
dihexosylceramides
lysoglycerophospholipids
monoacylglycerophosphoethanolamines [GP0205]
positive intrinsic curvature
mitochondrion
glycerophosphoethanolamines [GP02]
fatty acid with 24 carbons
C24:1
very high bilayer thickness
membrane component
lipid-mediated signalling
fatty acid with 26 carbons
fatty acid with more than 18 carbons
glycerophospholipids [GP]
fatty acid with more than 24 carbons
d18:1 (sphingosine)
very low lateral diffusion
C26:1
C26:0
endoplasmic reticulum (ER)
C22:1
above average bilayer thickness
C24:0
below average lateral diffusion
fatty acid with 22-24 carbons
fatty acid with less than 2 double bonds
monounsaturated fatty acid
C20:3
contains ether-bond
lkyl,2-acylglycerophosphoethanolamines [GP0202]
headgroup with neutral charge
C20:1
headgroup with positive charge / zwitter-ion
C22:0
Membrane Related
Unsaturation Related
Glycerolphospholipids
Others
B
Up in A172
Up in T98G

Figure S9. Lipid ontology enrichment and network analysis comparing A172 and T98G cells.

(A) Lipid ontology enrichment analysis of lipid species significantly enriched in A172 relative to T98G cells. Differentially abundant lipids were identified from untargeted LC–MS analysis (adjusted $p < 0.05$, $|\log2FC| \geq 1$) and analyzed using LION/web (Lipid Ontology Enrichment Analysis). Enrichment scores reflect overrepresentation of lipid ontology terms among significantly altered lipid species.

(B) Network visualization of enriched lipid ontology terms generated using LION/web. Nodes represent significantly enriched lipid ontology categories, and edges indicate relationships between ontology terms and associated lipid species. Node size corresponds to enrichment significance (–log10 adjusted p-value), and color reflects the direction of enrichment (higher in A172 vs. higher in T98G). The network highlights coordinated lipid remodeling patterns distinguishing less proliferative and more aggressive phenotypes.

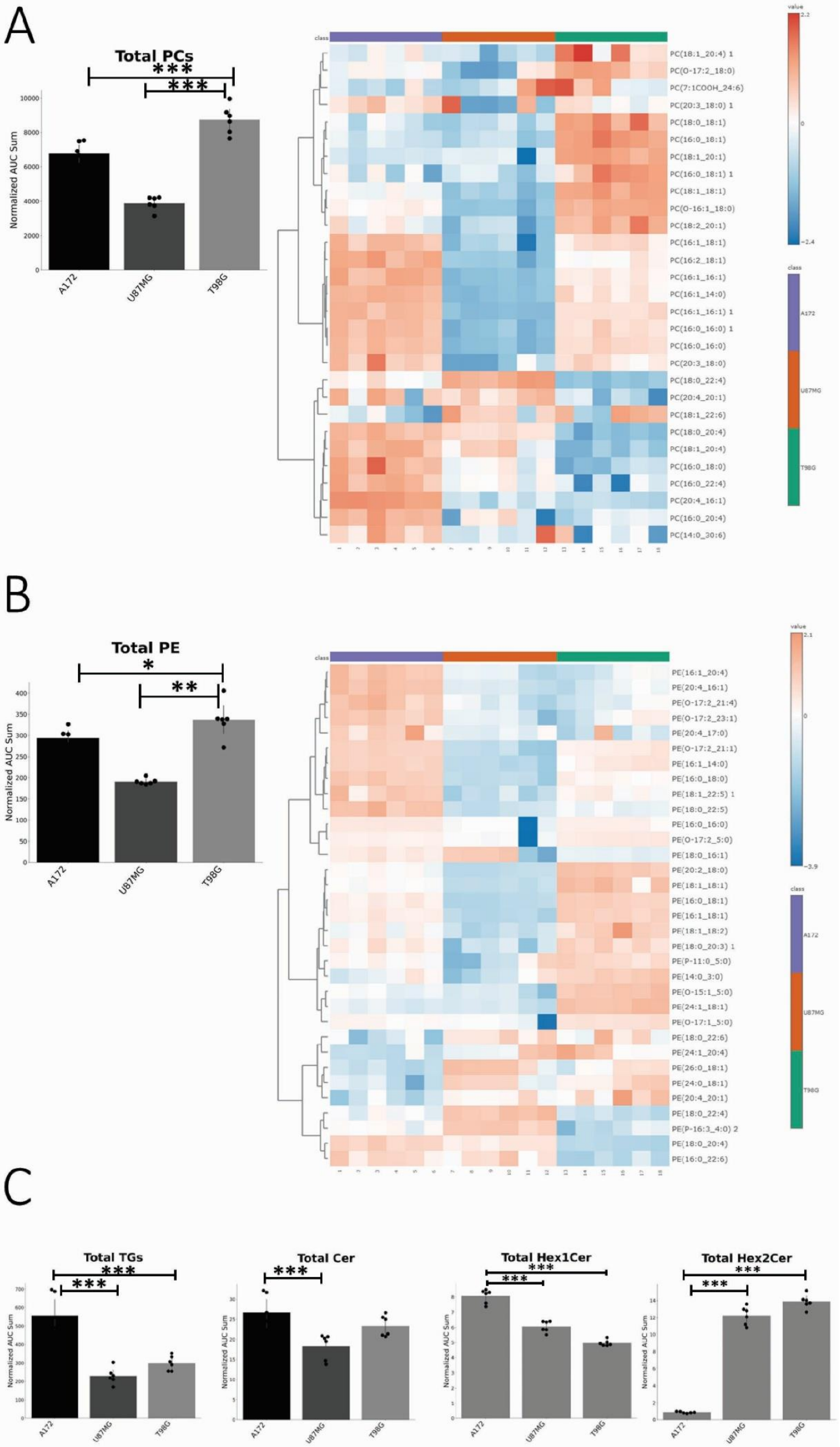
A
Total PCs
***
***
Normalized AUC Sum
A172
U87MG
T98G
class
value
PC(18:1_20:4) 1
PC(O-17:2_18:0)
PC(7:1COOH_24:6)
PC(20:3_18:0) 1
PC(18:0_18:1)
PC(16:0_18:1)
PC(18:1_20:1)
PC(16:0_18:1) 1
PC(18:1_18:1)
PC(O-16:1_18:0)
PC(18:2_20:1)
PC(16:1_18:1)
PC(16:2_18:1)
PC(16:1_16:1)
PC(16:1_14:0)
PC(16:1_16:1) 1
PC(16:0_16:0) 1
PC(16:0_16:0)
PC(20:3_18:0)
PC(18:0_22:4)
PC(20:4_20:1)
PC(18:1_22:6)
PC(18:0_20:4)
PC(18:1_20:4)
PC(16:0_18:0)
PC(16:0_22:4)
PC(20:4_16:1)
PC(16:0_20:4)
PC(14:0_20:6)
B
Total PE
*
**
Normalized AUC Sum
A172
U87MG
T98G
class
value
PE(16:1_20:4)
PE(20:4_16:1)
PE(O-17:2_21:4)
PE(O-17:2_23:1)
PE(20:4_17:0)
PE(O-17:2_21:1)
PE(16:1_14:0)
PE(16:0_18:0)
PE(18:1_22:5) 1
PE(18:0_22:5)
PE(16:0_16:0)
PE(O-17:2_5:0)
PE(18:0_16:1)
PE(20:2_18:0)
PE(18:1_18:1)
PE(16:0_18:1)
PE(16:1_18:1)
PE(18:1_18:2)
PE(18:0_20:3) 1
PE(P-11:0_5:0)
PE(14:0_3:0)
PE(O-15:1_5:0)
PE(24:1_18:1)
PE(O-17:1_5:0)
PE(18:0_22:6)
PE(24:1_20:4)
PE(26:0_18:1)
PE(24:0_18:1)
PE(20:4_20:1)
PE(18:0_22:4)
PE(P-16:3_4:0) 2
PE(18:0_20:4)
PE(16:0_22:6)
C
Total TGs
***
***
Total Cer
***
Total Hex1Cer
***
***
Total Hex2Cer
***
***
Normalized AUC Sum
A172
U87MG
T98G

Figure S10. Lipid class abundance and species-level remodeling in A172 and T98G cells.

(A) Total lipid class abundance comparing, phosphatidylcholines (PC), and Heatmap of differentially abundant PC species between cells. (B) Total lipid class abundance comparing phosphatidylethanolamines (PE), and Heatmap of differentially abundant PC species between cells. (C) Total lipid class abundance comparing and triacylglycerols (TG), ceramides (Cer) and hexosylceramides (HeCer and Hex2Cer) between cells. Class-level values represent the summed normalized abundance of all detected species within each lipid class. PE and PC levels were significantly higher in T98G cells, whereas TG abundance was increased in A172 cells. Data is from one biological replicate which is representative of three independent biological replicates per cell line and are presented as mean ± SD. Statistical significance was determined using T-test, with significance thresholds indicated in the figure, *p<0,05; **p<0,01;***p<0,001.

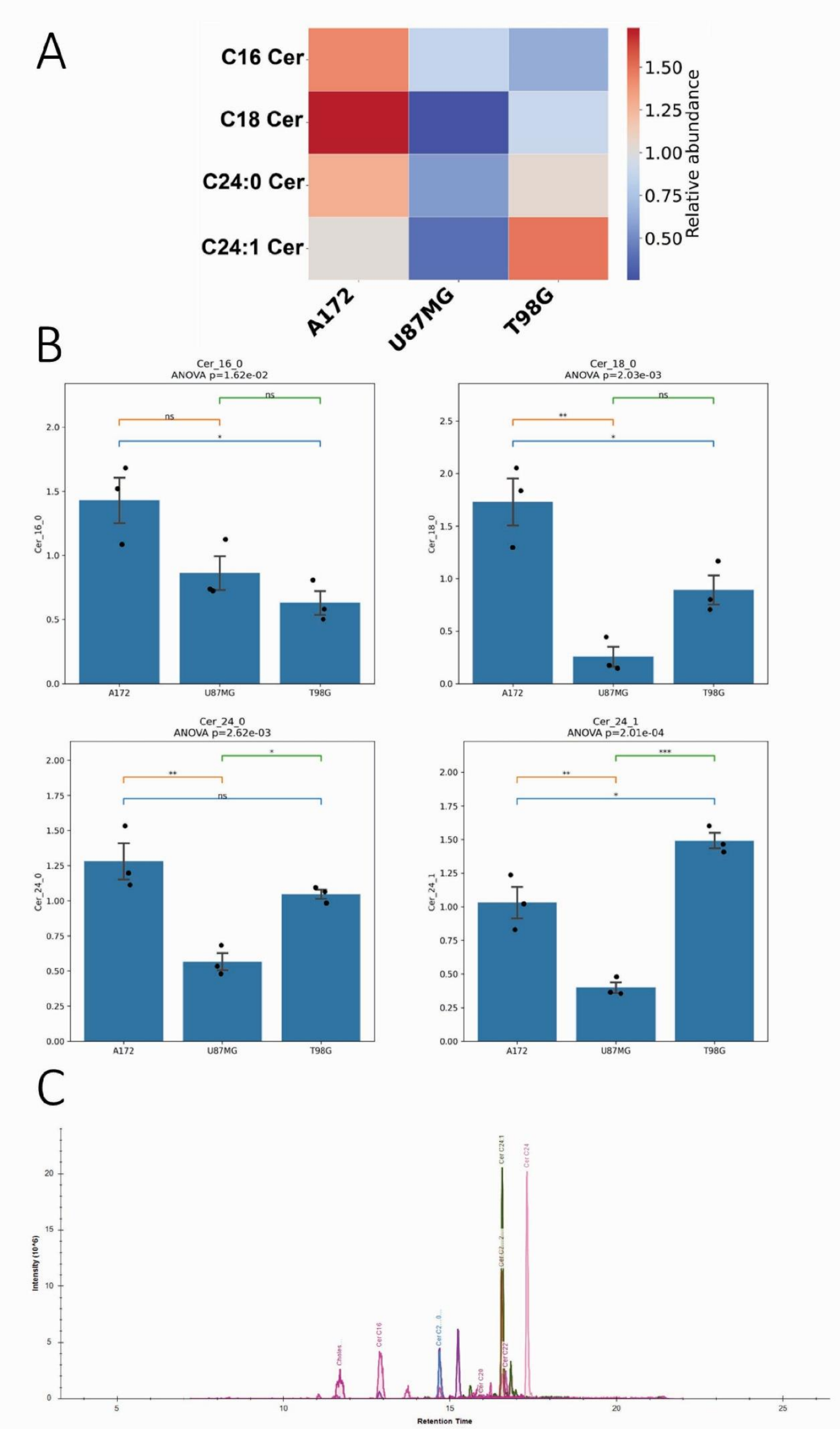

A
C16 Cer
C18 Cer
C24:0 Cer
C24:1 Cer
A172
U87MG
T98G
Relative abundance
1.50
1.25
1.00
0.75
0.50
B
Cer_16_0
ANOVA p=1.62e-02
Cer_18_0
ANOVA p=2.03e-03
Cer_24_0
ANOVA p=2.62e-03
Cer_24_1
ANOVA p=2.01e-04
ns
*
**
***
A172
U87MG
T98G
C
Intensity (10^6)
Retention Time
Cer C24:1
Cer C24
Cer C16
Cholles
Cer C22
Cer C20

Figure S11. Differential ceramide species distribution across glioblastoma cell lines. (A) Heatmap showing relative abundance of selected ceramide species (Cer16:0, Cer18:0, Cer24:0, and Cer24:1) in A172, T98G, and U87MG cells. Data represent normalized lipid abundance derived from targeted LC–MS analysis (n = 3 independent biological replicates per cell line). The heatmap highlights enrichment of long-chain ceramides (Cer16:0 and Cer18:0) in the less proliferative A172 cells and increased very-long-chain ceramides (particularly Cer24:1) in the more aggressive cell lines.

(B) Bar plots comparing relative abundance of individual ceramide species between cell lines. Data are presented as mean ± SD from three independent biological replicates. Statistical significance was determined using unpaired two-tailed Student's t-tests. Exact p-values are indicated in the figure, and significance thresholds are defined as *p < 0.05, **p < 0.01, and ***p < 0.001. (C) Representative extracted ion chromatograms (EICs) for selected ceramide species obtained from targeted LC–MS analysis. Chromatograms demonstrate peak separation, retention time consistency, and signal specificity for the quantified lipid species. Shown traces are representative of three independent biological replicates per cell line

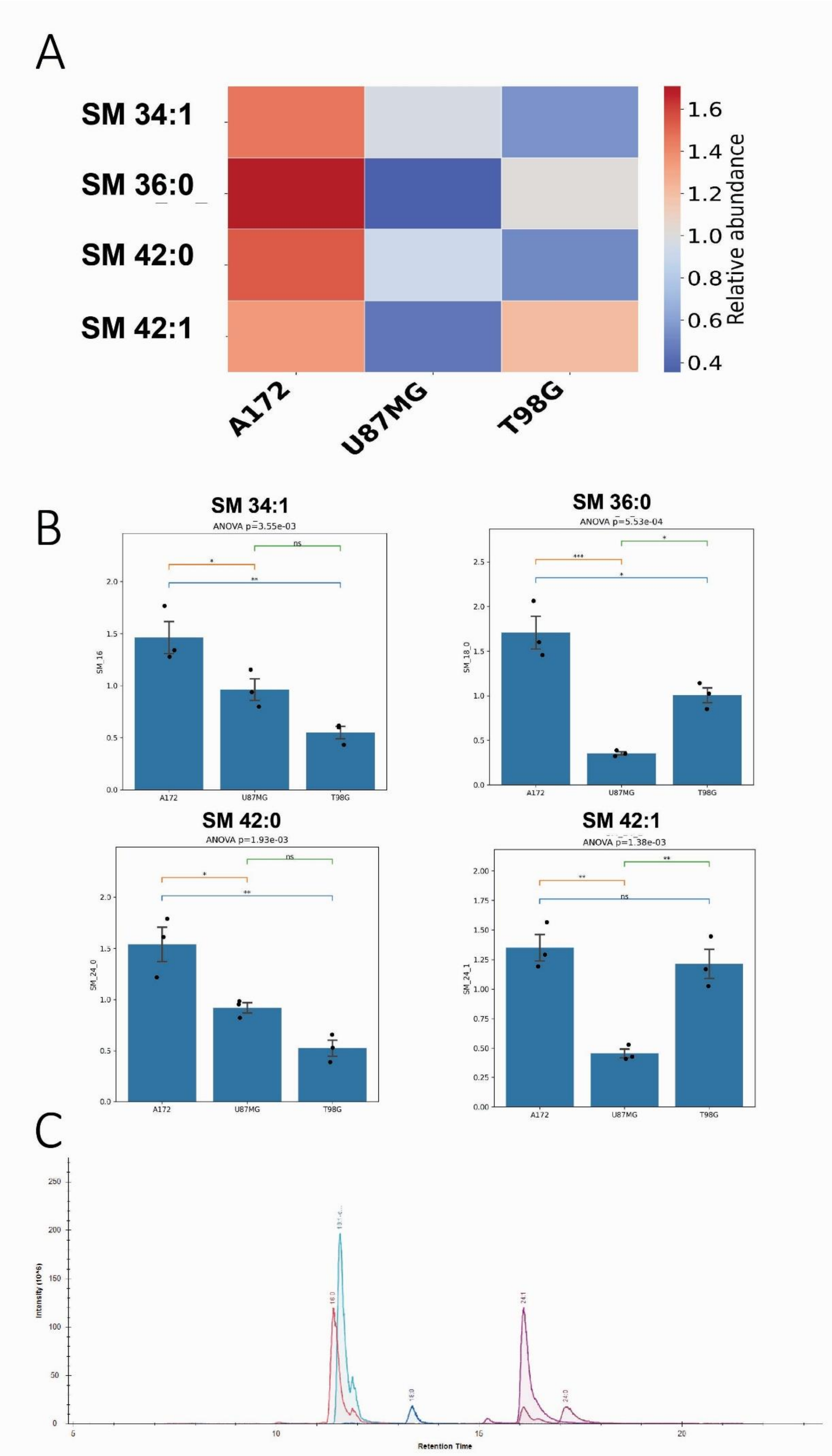
A
SM 34:1
SM 36:0
SM 42:0
SM 42:1
A172
U87MG
T98G
Relative abundance
1.6
1.4
1.2
1.0
0.8
0.6
0.4
B
SM 34:1
ANOVA p=3.55e-03
SM 36:0
ANOVA p=5.53e-04
SM 42:0
ANOVA p=1.93e-03
SM 42:1
ANOVA p=1.38e-03
SM_16
SM_18_0
SM_24_0
SM_24_1
ns
A172
U87MG
T98G
C
Intensity (10^6)
Retention Time
16:0
18:0
24:1
24:0

Figure S12. Differential sphingomyelin species distribution across glioblastoma cell lines.

(A) Heatmap showing relative abundance of selected sphingomyelin species (SM16:0, SM18:0, SM24:0, and SM24:1) in A172, T98G, and U87MG cells. Data represent normalized lipid abundance obtained from targeted LC–MS analysis (n = 3 independent biological replicates per cell line. The heatmap illustrates differential distribution of long-chain and very-long-chain sphingomyelin species across cell lines.

(B) Bar plots comparing relative abundance of individual sphingomyelin species between cell lines. Data are presented as mean ± SD from three independent biological replicates. Statistical significance was determined using unpaired two-tailed Student's t-tests. Exact p-values are indicated in the figure, with significance thresholds defined as $*p < 0.05$, $**p < 0.01$, and $***p < 0.001$. (C) Representative extracted ion chromatograms (EICs) for selected sphingomyelin species obtained from targeted LC–MS analysis. Chromatograms demonstrate peak separation, retention time consistency, and signal specificity for the quantified lipid species. Shown traces are representative of three independent biological replicates per cell line

Supplementary Table 1 – Coefficients of genes selected to calculate the risk score.

Gene	Coefficients
ACAA2	-0.0935
AHR	-0.1006
ANGPTL4	0.1411
ARSD	0.1821
CYP27B1	0.1714
FHL2	-0.0508
GLB1	0.5115
GLIPR1	0.0907
MBOAT1	0.1402
OSBPL10	0.0698
PIK3CG	0.2923
PIK3R6	-0.0645
PLA2G4A	0.1130
PLEKHA4	0.1025
PNPLA4	0.1472
PTGS2	-0.0608
SEC24D	-0.1531
TSPO	-0.0842
UGCG	-0.0364
CYP21A2	-0.0178
FASN	0.1210
HMGCS1	-0.1421
NEU4	-0.0021
PLEKHA5	-0.1042
PLEKHA6	0.1062
PPM1L	-0.0743
SCD	-0.1464
SLCO1A2	-0.0629
SMPD3	0.0451

Supplementary Table 2 – ^{1}H NMR assignments performed in the organic extracts of the cell lines.

Table S2. The bold assignments refer to specific resonance in the fatty acid chain. Chol, Cholesterol; F_{β}, protons adjacent to the COOH group in the fatty acid chain; F_{α}, protons adjacent to the COOH group in the fatty acid chain; PE, Glycerophosphoethanolamines; PC, Glycerophosphocholines; PI, Glycerophosphoinositols; PS, Glycerophosphoserines; DG, Diacylglycerol; Cer, Ceramide; TG, Triacylglycerol; UFA, Unsaturated fatty acid.

Lipid Class	^{1}H Chemical shift
Chol-C18	0.6931
Chol-C19	1.0157
F_{β}:R-**CH_2**-CH_2-CO-	1.609
-CH=CH-**CH_2-** (18:1)	2.0202
-CH=CH-**CH_2**-CH=CH- (18:2;20:4)	2.0554
-CH=CH-CH_2-CH_2-**CH_2**-CO- (20:4)	2.1283
F_{α}: R-CH_2-**CH_2**-CO-	2.3256
-CH=CH-**CH_2**-**CH_2**-CO- (22:6)	2.4022
-CH=CH-**CH_2**-CH=CH- (18:2)	2.7765
-CH=CH-(**CH_2**-CH=CH-)$_y$ (20:4,22:6)	2.8142
-$N^+(Me_3)_3$ (PC)	3.2163
PC-2’	3.5861
Gly-C3 (DG)	3.6998
Gly-C1 (DG/TG/PC/PE/PI)	4.1665
PC-1’	4.2431
Gly-C2 (DG)	5.1694
Gly-C2 (PC/PE/PI)	5.2301
Mono-UFA	5.3492
Poly-UFA	5.3724
